# Interaction between the RNA-dependent ATPase and poly(A) polymerase subunits of the TRAMP complex is mediated by short peptides and important for snoRNA processing

**DOI:** 10.1093/nar/gkv005

**Published:** 2015-01-14

**Authors:** Jillian S. Losh, Alejandra Klauer King, Jeremy Bakelar, Lacy Taylor, John Loomis, Jason A. Rosenzweig, Sean J. Johnson, Ambro van Hoof

**Affiliations:** 1 Department of Microbiology and Molecular Genetics, University of Texas Health Science Center-Houston, Houston, TX 77030, USA; 2 The University of Texas Graduate School of Biomedical Sciences at Houston, Houston, TX, USA; 3 Department of Chemistry and Biochemistry, Utah State University, Logan, UT 84322–0300, USA; 4 Department of Biology and Department of Environmental and Interdisciplinary Sciences, Texas Southern University, Houston, TX 77004, USA

## Abstract

The RNA exosome is one of the main 3′ to 5′ exoribonucleases in eukaryotic cells. Although it is responsible for degradation or processing of a wide variety of substrate RNAs, it is very specific and distinguishes between substrate and non-substrate RNAs as well as between substrates that need to be 3′ processed and those that need to be completely degraded. This specificity does not appear to be determined by the exosome itself but rather by about a dozen other proteins. Four of these exosome cofactors have enzymatic activity, namely, the nuclear RNA-dependent ATPase Mtr4, its cytoplasmic paralog Ski2 and the nuclear non-canonical poly(A) polymerases, Trf4 and Trf5. Mtr4 and either Trf4 or Trf5 assemble into a TRAMP complex. However, how these enzymes assemble into a TRAMP complex and the functional consequences of TRAMP complex assembly remain unknown. Here, we identify an important interaction site between Mtr4 and Trf5, and show that disrupting the Mtr4/Trf interaction disrupts specific TRAMP and exosome functions, including snoRNA processing.

## INTRODUCTION

Almost all cellular RNAs undergo extensive processing before becoming fully mature and functional RNAs. The pathways involved in these post-transcriptional modifications, although tightly regulated, are not error-free. Therefore, cells have evolved a variety of surveillance mechanisms to ensure the fidelity of this RNA maturation process and gene expression. The RNA exosome complex is a major player in both RNA processing and surveillance mechanisms. For example, the RNA exosome has 3′ to 5′ exoribonuclease activity that is required for removal of 3′ extensions from precursor RNAs (e.g. 5.8S rRNA; 1,2) and to degrade misprocessed RNAs (e.g. hypomethylated tRNAi; 3). Many exosome substrates have been identified, but how the exosome distinguishes between substrates that require processing, substrates that require degradation and non-substrates is poorly understood.

One determinant of exosome specificity appears to be provided by over a dozen proteins that are collectively called exosome cofactors. These proteins are thought to initiate interaction with substrates and deliver them to the exosome by poorly defined mechanisms. Most prominent among these cofactors are two RNA-dependent ATPases (also called RNA helicases), Mtr4 and Ski2 (4,5). Unlike other exosome cofactors that appear to be specific for a limited number of exosome functions, Mtr4 is required for all known nuclear exosome functions, while Ski2 is required for all known cytoplasmic exosome functions.

Mtr4 is a subunit of the heterotrimeric Trf4/5 Air1/2 Mtr4 polyadenylation (TRAMP) complex. In the yeast *Saccharomyces cerevisiae*, the other two subunits are one non-canonical poly(A) polymerase (either Trf4 or Trf5), and one zinc knuckle RNA binding protein (either Air1 or Air2; 6,7). The duplicated *TRF* and *AIR* genes arose during a whole genome duplication in an ancestor of *S. cerevisiae* (http://ygob.ucd.ie/; 8,9) and, therefore, other eukaryotes have only one ortholog of each, which in humans are named PAPD5 and ZCCHC7, respectively. Furthermore, it has been shown that the hMTR4, PAPD5 and ZCCHC7 proteins interact (10), suggesting that TRAMP complex formation is conserved between fungi and animals. In yeast, Mtr4 is encoded by an essential gene, but *TRF4, TRF5, AIR1* and *AIR2* are not individually essential (4,11). However, a *trf4Δ trf5Δ* double mutant is inviable (12), and an *air1Δ air2Δ* double mutant is extremely slow growing (13). These growth phenotypes suggest that all three subunits of the TRAMP complex perform critical functions, although the importance of assembly into a TRAMP complex is not understood.

Importantly, Mtr4 is more abundant than the other TRAMP subunits, indicating that it exists outside TRAMP, and some functions of the exosome require Mtr4 but not the other TRAMP subunits (6). The TRAMP complex is thought to be required for degrading incorrectly processed RNAs, including rRNAs and tRNAs, as well as non-coding RNAs with unknown functions (cryptic unstable transcripts and stable unannotated transcripts; 14,15). An example of a reaction that requires Mtr4, but not other TRAMP subunits is the processing of 5.8S rRNA from a 3′ extended precursor (6). One hypothesis is that TRAMP is involved in exosome-mediated RNA degradation, while TRAMP-independent Mtr4 is involved in exosome-mediated RNA processing. Definitive proof for this hypothesis would require showing whether or not 3′ extended and/or polyadenylated species that accumulate in TRAMP mutants are precursors in the normal processing pathway, or aberrant transcripts marked for degradation.

Adding nucleotides to the 3′ end of an RNA to facilitate removal of nucleotides in the 3′ to 5′ direction is somewhat counterintuitive, but several roles of TRAMP-mediated polyadenylation in exosome-mediated degradation have been offered. First, the main catalytic subunit of the RNA exosome (Rrp44) is accessed through a long narrow central channel formed by the other nine subunits of the exosome (16,17). Therefore, exosome-mediated degradation is thought to require a long unstructured region, which may be provided by TRAMP-mediated polyadenylation. Under this hypothesis, the unstructured tail would have to be long enough to traverse the channel (∼30 nts). Such long TRAMP-dependent tails have been detected in exosome mutants and TRAMP can synthesize long A-tails *in vitro* (6,7,18,19). However, a typical TRAMP-synthesized tail in wild-type cells is thought to be only 3–4 nts (20,21) and, thus, not long enough to completely pass through the exosome central channel. One caveat is that the short A-tails seen in wild-type cells are necessarily the ones that have not (yet) been degraded by the exosome. RNAs that receive longer A-tails may be more rapidly degraded by the exosome. Therefore, the short A-tails seen at steady state may not be representative of all the tails synthesized by TRAMP. Another indication that long, unstructured 3′ tails are not required for exosome-mediated degradation comes from the observation that the exosome appears to be fully capable of degrading cytoplasmic substrate RNAs independently of a poly(A) polymerase, including substrates that contain very stable secondary structures (e.g. G-quadruplexes; 22,23). An alternative hypothesis is based on the observation that Mtr4 specifically interacts with oligo-adenylated RNAs (24–26), and thus TRAMP-synthesized tails may target substrates to Mtr4 rather than directly to the RNA exosome. Under both of these hypotheses, substrate RNAs would initially interact with the poly(A) polymerase subunit of TRAMP, and subsequently be handed off to the exosome. Strikingly, while these hypotheses explain the role of the poly(A) tail, neither of these hypotheses readily explain why Mtr4 and a poly(A) polymerase assemble into a TRAMP complex. A third possibility is that polyadenylation by Trf4/5 occurs in response to a block or stall during normal processing by the exosome. Physical interaction between the Mtr4/exosome machinery and Trf4/5 may facilitate polyadenylation of such degradation product and polyadenylation may enhance subsequent re-engagement of the Mtr4/exosome machinery. Consistent with the idea that TRAMP can act on partially degraded RNAs is that TRAMP-dependent tails are added at multiple positions, including the region corresponding to the mature RNA (27). Thus, it is not clear whether stable association of the poly(A) polymerase with Mtr4 facilitates substrate hand-off from the Mtr4/exosome machinery to the poly(A) polymerase or *vice versa*.

Resolving these questions on TRAMP and Mtr4 function requires a better understanding of how the subunits of TRAMP interact and of the consequences of impairing TRAMP complex assembly. Here, we identify a short peptide on the poly(A) polymerase subunit that is important for TRAMP complex assembly. This short peptide is essentially the only conserved sequence in the N-terminus of the poly(A) polymerase subunit. Furthermore, this N-terminus is predicted to be largely disordered. We also show that deletion of this peptide impairs assembly of TRAMP and has a specific effect on the accumulation of 3′ extended snoRNAs. We anticipate that the strain we generated will be an important tool in future characterization of TRAMP function.

## MATERIALS AND METHODS

### Plasmids

The yeast two-hybrid assays used plasmids pOBD2 and pOAD that were previously described (28). The yeast two-hybrid plasmids for Mtr4 (pAV745), *Mtr4-archless* (pAV746) and *Trf5–53–199* (pAV744) have been previously described as have the positive controls that contain *MEC3* and *RAD17* (29). Yeast two-hybrid plasmids with Trf5 residues 68–199 (pAV759), 83–199 (pAV760), 98–199 (pAV761), 118–199 (pAV766), 53–184 (pAV762), 53–169 (pAV763), 53–154 (pAV764), 53–139 (pAV767) and 53–124 (pAV768) were generated similarly to pAV744. Briefly, these regions of *TRF5* were PCR amplified with primers that contain PvuII and PstI sites, digested with those restriction enzymes and cloned into pOAD. The yeast two-hybrid plasmids with Trf5 residues 98–117 were generated by cloning oligonucleotides for this region into the PvuII and PstI sites of pOAD.

The Trf5-Δ98–117-TAP and Trf5-TAP plasmids are low copy (CEN) plasmids derived from pRS415 (30) with the endogenous TRF5 promoter driving expression of Trf5 or Trf5*-Δ98–117*. The *trf5-Δ98–117-TAP* plasmid (pAV854) was generated by ligation of three DNA fragments. The first fragment contained the promoter and 5′ end of *TRF5* and was generated by PCR with oligonucleotides oAV906 (TATTATGCGGCCGCCCACAAAGTACTACATCTATGGTCT) and oAV870 (GCGGCGACTAGTTCTTTTGCTAGATTCTGCCCTTTGTTC) and digestion with NotI and SpeI. The second fragment contained the 3′ end of *TRF5* and was generated by PCR with oligonucleotides oAV871 (GCGGCGACTAGTGAACAAATAAAGGAAGATGATGATG) and oAV869 (GCGGCGCTGCAGCAAGAGCCTGGCCTTTAGAGAGCC) and digestion with SpeI and PstI. The third fragment was pAV476 (31) digested with NotI and PstI. The *TRF5-TAP* plasmid (pAV885) was subsequently generated by replacing an XbaI to BstAPI fragment that contained the internal deletion with the corresponding wild-type sequence.

Cloning of full-length Mtr4^WT^ (pSJJ004) and Mtr4^archless^ (pSJJ009) expression constructs was performed previously (32). Additional truncated variants used in this study include Mtr4^Δ74^ (pSJJ012, comprising residues 75–1073), Mtr4^1–614^ (pSJJ014) and Mtr4^665–815^ (pSJJ020). Each of the Mtr4 constructs was cloned into a pET151/D-TOPO vector with a TEV protease-cleavable 6-His tag on the N-terminus.

### Yeast strains

Strain *trf4Δ/trf5Δ/pRS416-TRF4* has been previously described (*MATa, leu2-Δ0, ura3-Δ0, his3-Δ1, met15-Δ0, trf4Δ::NAT, trf5Δ::KAN*; 33). The *TRF5* and *trf5-Δ98–117* plasmids were introduced by transformation and selection on media lacking leucine. Transformants were then plated on media containing 5FOA to select for cells that had lost the pRS416-TRF4 plasmid. The r*rp44-exo^−^, rrp44-endo^−^* and *rrp6Δ::KAN* strains are isogenic to this strain and have been previously described (31). Yeast two-hybrid strains PJ69–4α and PJ69–4a have been previously described (28,34). They are MAT*α* and *MATa*, respectively, and in addition are both *trp1–901, leu2–3*,*112, ura3–52, his3–200, gal4Δ, gal80Δ, LYS2::GAL1-HIS3, GAL2-ADE2, met2::GAL7-lacZ.*

### Yeast two-hybrid assays

Yeast two-hybrid assays were carried out as previously described (29). Briefly, the *MTR4 TRP1* plasmids were transformed into PJ69–4α and the *TRF5 LEU2* plasmids were transformed into PJ69–4a. Transformants were crossed to each other and diploids that contain both plasmids were selected on SC-TRP-LEU. These diploids were then serially diluted and spotted on SC-TRP-LEU, SC-ADE and SC-HIS media.

### Co-purification assays

The purification of TAP-tagged proteins was done as previously described (22). Briefly, yeast cells were lysed by vortexing in the presence of glass beads and IP50 buffer (50 mM TRIS HCl pH7.5, 50 mM KCl, 2 mM MgCl_2_, 0.1% triton X100 and cOmplete EDTA-free protease inhibitors; Roche). The lysate was clarified by centrifugation and incubated with IgG Sepharose (GE Healthcare) for 2 h at 4°C. The beads were pelleted, washed twice with IP50 and twice with IP150 (same as IP50, except 150 mM KCl instead of 50 mM), before being mixed with Laemmli loading buffer. Western blot analysis used antibodies against protein A (Sigma), Mtr4 (Gift from Patrick Linder) and Pgk1 (Invitrogen).

### RNA analysis

RNA isolation and northern blotting was performed essentially as previously described (32). Quantitative reverse transcriptase-PCR (qRT-PCR) was carried out using the SYBR Green RNA-to-Ct 1-Step kit from Applied Biosystems as per the manufacturer's instructions with primers that were previously described (15). Transcriptome sequencing was performed by LC Sciences (Houston, TX, USA) on a HiSeq 2500. Briefly, poly(A)^+^ RNA was isolated from duplicate cultures of the *trf4Δ, trf5Δ* deletion strain carrying either the *TRF5* or *trf5-Δ98–117* plasmid and converted to a sequencing library. Each library was sequenced, yielding between 10 and 14 million 50 nt reads that were mapped to the annotated yeast genes (ftp://ftp.ensembl.org/pub/release-77/fasta/saccharomyces_cerevisiae/dna/) using Bowtie. Genes that were significantly up- or down-regulated in the *trf5-Δ98–117* mutant were identified using edgeR (35).

### Recombinant protein expression and purification

The expression and purification of Mtr4^WT^ and truncated Mtr4 proteins was carried out as performed previously (32). All proteins were recombinantly expressed in an *Escherichia coli* BL21-codon+-(DE3)-RIL cell line (Stratagene). Cell lysis was performed by lysozyme treatment and sonication of frozen cell pellets. Nickel affinity, Heparin affinity and Superdex 200 (GE) gel filtration was used to purify Mtr4 and truncated Mtr4 variants. Final purification buffer consisted of 50 mM HEPES (pH 7.5), 160 mM NaCl, 5% glycerol and 2 mM β-mercapto-ethanol.

### Fluorescence anisotropy

Binding analysis of Mtr4 to Trf5 and Air2 was carried out using fluorescence anisotropy. A Trf5 peptide comprising residues 98–124 with an N-terminal fluorescein label was purchased through the Keck lab at Yale University. Binding reactions were buffered in 50 mM HEPES (pH 7.5), 140 mM NaCl and 12% glycerol. Concentration of the fluorescently labeled peptide was held constant at 50 nM, with increasing concentrations of Mtr4. Mtr4 was incubated with the fluorescein-labeled Trf5 peptide for 30 min at 30°C prior to collection of fluorescence anisotropy data. Anisotropy was measured using a Synergy H4 Hybrid Multi-Mode Microplate Reader (BioTek) with an excitation at 485 ± 20 nm and an emission at 528 ± 20 nm (the appropriate filters for detection of fluorescein) at 30°C.

For Air2 binding studies, an Air2 peptide comprising residues 1–29 with an N-terminal fluorescein label was obtained from Dr. Joshua Price at Brigham Young University. Binding reactions were buffered in 50 mM HEPES (pH 7.5), 100 mM NaCl, 0.1 mg/ml bovine serum albumin and 12% glycerol. Concentration of the labeled Air2^1–29^ peptide was held constant at 80 nM and titrated with increasing concentrations of Mtr4. Samples were incubated for 3 min after each titration, as changes in anisotropy were not observed beyond this incubation time. Anisotropy at each titration point was measured 10 times and averaged. Anisotropy measurements were obtained using a steady-state-photon counting spectrofluorometer, PC1 with Vinci software, from ISS Instruments. Excitation and emission slits were adjusted to 0.5 nm and temperature was maintained at 25°C. The excitation wavelength was 495 nm and emission anisotropy was measured at 521 nm.

## RESULTS

### A conserved 20 amino acid peptide in the N-terminus of Trf5 is sufficient for Mtr4 interaction

**Figure 1.**
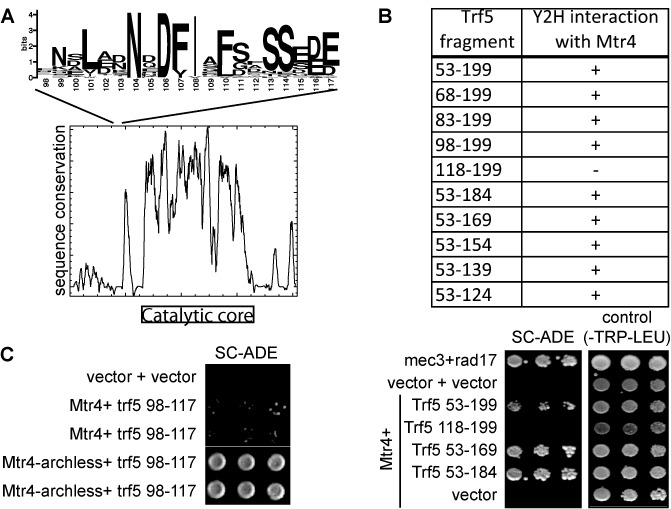
A conserved 20 amino acid peptide in the N-terminus of Trf5 is sufficient for Mtr4 interaction. (**A**) Sequences of Trf4 and Trf5 of 25 different yeast species were aligned, revealing limited sequence conservation outside the catalytic core. Highlighted above the graph is a conserved sequence motif in the otherwise poorly conserved N-terminus. The numbering below the motif (98–117) reflects the amino acid numbering in Trf5. (**B**) Yeast two-hybrid analysis identifies amino acids 98–117 of Trf5 as critical for interaction with Mtr4. The table summarizes the yeast two-hybrid assays performed. ‘+’ indicates growth on both SC-ADE and SC-HIS supplemented with 10 mM 3-AT. ‘-’ indicates a failure to interact. Representative results for some constructs are included below the table. Mec3 and Rad17 are known to interact with each other and were included as positive controls. (**C**) Yeast two-hybrid interaction indicates amino acids 98–117 of Trf5 are sufficient for interaction with Mtr4. Duplicate analyses are shown in C.

The TRAMP complex was initially identified in a yeast two-hybrid screen for Mtr4 interacting proteins (6). All of the Trf5 clones identified included amino acid residues 53–199, suggesting that this part of Trf5 contains the Mtr4 interacting site. Bioinformatic analysis did not reveal any recognizable domains contained within this region, but PONDR (Predictor of Naturally Disordered Regions; http://www.pondr.com/index) suggested that this region was largely intrinsically disordered. Multiple sequence alignment showed that, similar to other intrinsically disordered regions, the sequence was generally poorly conserved, except for residues 98–117 of Trf5. As shown in Figure 1A and Supplementary Figure S1, this 20 amino acid region is conserved in yeast Trf4 and Trf5 proteins. Since small conserved motifs in intrinsically disordered regions are often protein–protein interaction motifs (reviewed in 36), we hypothesized that this may be a major Mtr4 interacting site.

To test whether amino acids 98–117 are sufficient for a yeast two-hybrid interaction with Mtr4, we generated further truncations starting with the Trf5 53–199 fragment identified previously (6). Deleting 15, 30 or 45 amino acids from the N-terminus of the 53–199 region did not affect yeast two-hybrid interaction with Mtr4 (Figure 1B), suggesting the main interaction site was C-terminal of amino acid 98. In contrast, deleting 60 amino acids from the N-terminus abrogated the yeast two-hybrid interaction. In similar C-terminal deletions, deleting 15, 30, 45, 60 or 75 amino acids from the 53–199 region did not affect yeast two-hybrid interaction with Mtr4, suggesting the main interaction site was N-terminal of amino acid 124 (Figure 1B). Thus, these results are consistent with the conserved peptide of amino acid residues 98–117 forming a major Mtr4 interaction site.

To more directly test whether amino acids 98–117 of Trf5 form an Mtr4-interaction site, we cloned just these residues into a yeast two-hybrid vector. As shown in Figure 1C, this peptide indeed interacted with Mtr4. We and others have previously solved the crystal structure of Mtr4, which revealed that it consists of a core helicase fold shared with the Ski2-like family of RNA and DNA helicases and an arch domain that is present only in Mtr4, Ski2 and their orthologs (32,37). Deleting this arch domain did not disrupt the interaction with other TRAMP subunits (32,37). Similarly, the 98–117 peptide of Mtr4 interacts both with full-length and archless Mtr4 (Figure 1C). In fact, in the yeast two-hybrid analysis, the Mtr4-archless interaction with Trf5 98–117 allowed for more robust growth, as we have previously reported for the longer Trf5 53–199 fragment. These results identify an interaction of a small peptide in the unstructured N-terminus of Trf4/5 with the helicase core of Mtr4 that may be important for TRAMP complex assembly.

**Figure 2.**
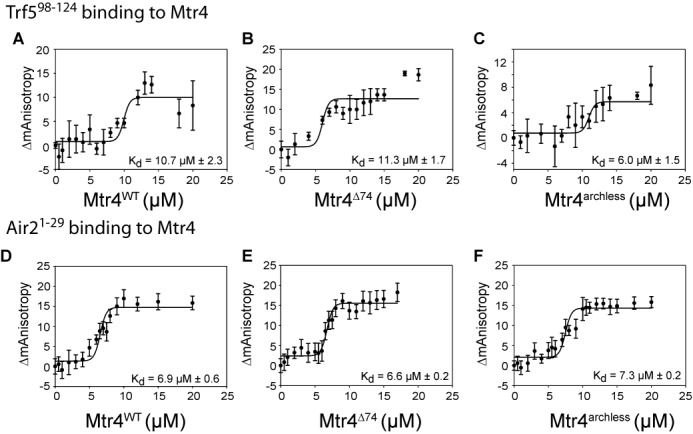
A conserved 20 amino acid peptide in the N-terminus of Trf5 interacts with the Mtr4 core *in vitro*. Shown is fluorescence anisotropy analysis of a Trf5 peptide binding to full-length recombinant Mtr4 (**A**), Mtr4 with the unstructured N-terminus deleted (**B**) or Mtr4 with the arch domain deleted (**C**). (**D–F**) Similar analyses for an Air2 peptide.

To test whether Trf5 residues 98–117 directly bind to purified recombinant Mtr4, we used fluorescence anisotropy. Figure 2A shows that indeed, a fluorescently labeled Trf5 peptide directly bound to recombinant Mtr4 with a *K_d_* of 10.7 μM. This binding was specific as no binding was observed of this peptide to purified recombinant Rrp47 (another exosome cofactor; data not shown). We further probed the Mtr4-Trf5 peptide interaction with truncated forms of Mtr4. An N-terminal truncation (Mtr4^Δ74^; Figure 2B), and an Mtr4 arch deletion (Mtr4^archless^; Figure 2C) both had binding affinities for the Trf5 peptide similar to full-length Mtr4. We conclude that the Trf5 peptide binds to the core region of Mtr4, consistent with a recent co-crystal structure (see Discussion; 38).

Although these fluorescence anisotropy data indicate that Trf5 residues 98–117 directly bind to Mtr4, the modest *K_d_* of 10.7 μM suggests other residues may also contribute. One candidate is the very N-terminus of the Air1/2 subunit, since previous co-immunoprecipitation experiments have implicated it in Mtr4 interaction (39). We tested a peptide corresponding to Air2 residues 1–29 for binding to Mtr4 by fluorescence anisotropy and observed an apparent *K*_d_ of ∼6.9 μM (Figure 2D). The Air2 peptide binds with similar affinity to an N-terminal truncation (Mtr4^Δ74^; Figure 2E) or an Mtr4 arch deletion (Mtr4^archless^; Figure 2F). Thus, while the Trf5–98–117 peptide can bind Mtr4, an Air1/2 peptide may also contribute to TRAMP complex assembly (see Discussion; 38,39).

### A 20 amino acid peptide in the N-terminus of Trf5 is important for TRAMP complex formation

**Figure 3.**
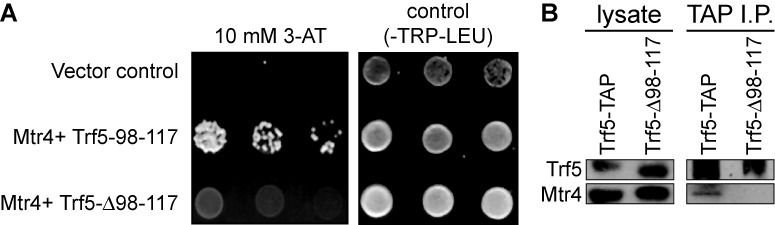
Amino acids 98–117 of Trf5 are required for interaction with Mtr4. (**A**) Trf5 98–117 interacts with Mtr4 in a yeast two-hybrid experiment, but Trf5-Δ98–117 does not. (**B**) Purification of full-length TAP-tagged Trf5 with IgG Sepharose results in co-purification of endogenous Mtr4, but this co-purification is disrupted by deleting amino acids 98–117.

Although the above analysis suggests two peptides mediate the interaction between Trf/Air and Mtr4, it does not address whether both are required. Since it has previously been shown that the Air N-terminal peptide is required for co-immunoprecipitating Mtr4, but not for viability (39), we focused on testing whether the Trf5 peptide is also important. Initially, we compared the binding of Mtr4 to either the Trf5 98–117 peptide or Trf5 deleted for this peptide by yeast two-hybrid analysis. Figure 3A shows that unlike Trf5 98–117, the Trf5 construct lacking 98–117 (Trf5-Δ98–117) fails to interact.

Although a positive interaction in yeast two-hybrid analysis is often informative, the negative interaction between Trf5-Δ98–117 and Mtr4 is not definitive. We therefore sought to analyze whether residues 98–117 are indeed required for Mtr4 interaction in the endogenous TRAMP complex. To this end, we generated TAP-tagged versions of both full-length Trf5 and Trf5-Δ98–117 and expressed these in yeast from their endogenous promoters. The TAP-tagged Trf5 proteins were purified and tested for co-purification with endogenous Mtr4 by western blot with antibodies raised against Mtr4. As shown in Figure 3B, endogenous Mtr4 was readily detectable in the purification of full-length Trf5, but not in the purification of Trf5-Δ98–117. We conclude that residues 98–117 of Trf5 form a major interaction site for Mtr4, and that preventing this interaction impairs formation of TRAMP *in vivo*.

### The Mtr4/Trf interaction is not required for viability, but is required for specific TRAMP functions

**Figure 4.**
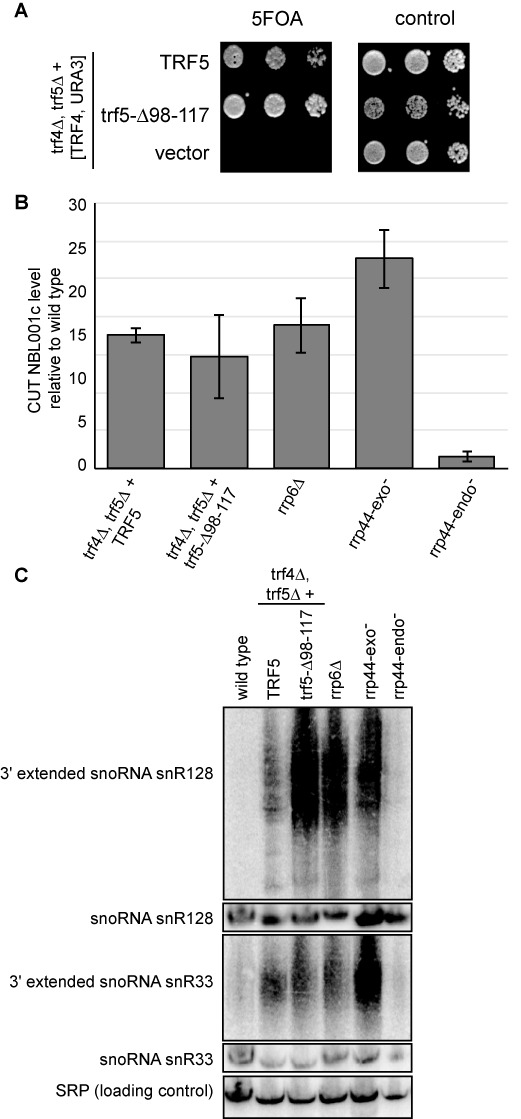
Disrupting stable TRAMP complex formation does not affect viability, but does result in some RNA processing defects. (**A**) The *trf5-Δ98–117* allele supports wild-type growth rates. A strain with deletions of both *TRF4* and *TRF5*, containing a wild-type *TRF4* gene on a *URA3* plasmid was transformed with *TRF5, trf5-Δ98–117* or empty *LEU2* plasmids. Transformants were serially diluted and spotted on 5FOA containing media or control SC-LEU media. Growth on 5FOA containing media indicates the *trf5-Δ98–117* allele is viable. (**B**) Steady-state levels of CUTs are not altered by interfering with assembly of a stable TRAMP complex. The level of CUTs was determined using qRT-PCR and is plotted relative to the level detected in a wild-type strain. The CUT levels are similar between the *TRF5* and *trf5-Δ98–117* strains. Similar results for two other CUTs not shown. (**C**) Steady-state levels of 3′ extended snoRNAs are increased by interfering with assembly of a stable TRAMP complex. Northern blots were probed with probes specific for the indicated species.

To explore the role of assembly of Trf4/5 with Mtr4 in a stable TRAMP complex, we tested whether the *trf5-Δ98–117* plasmid could function as the sole source of Trf4/5 through a plasmid shuffle assay. Surprisingly, the *trf5-Δ98–117* allele fully complemented the lethality of a *trf4Δ, trf5Δ* strain (Figure 4A). Furthermore, the steady-state protein levels of *Trf5-Δ98–117* were similar to those for wild-type Trf5 (data not shown). We conclude that stable association with Mtr4 is not needed for protein stability or for the essential function of Trf4/5.

Having generated a strain impaired for TRAMP complex formation, we tested whether it affected specific TRAMP functions. As their name implies, cryptic unstable transcripts (CUTs) are not readily detectable in wild-type cells, but they accumulate in mutants impaired in TRAMP or exosome activity (15). TRAMP is thought to be responsible for both their polyadenylation and subsequent degradation. We therefore used qRT-PCR with gene-specific primers during the reverse transcription step such that defects in either polyadenylation or subsequent degradation would be detectable. As expected, we detected accumulation of several different CUTs in the absence of either the exoribonuclease activity of Rrp44 or Rrp6 (*rrp44-exo^−^* and *rrp6Δ*, respectively; Figure 4B; data not shown for other CUTs; 15,40). In contrast, a strain lacking the endonuclease activity of Rrp44 (*rrp44-endo^−^*) did not accumulate increased CUT levels. Similarly, as previously reported (15,41,42), we detected CUT accumulation in the absence of Trf4, that is in *trf4Δ, trf5Δ* complemented with a full-length Trf5 plasmid. Importantly, the CUT steady-state levels in the *trf4Δ, trf5Δ* strain complemented with a Trf5-Δ98–117 plasmid were similar to those in the same strain complemented with full-length Trf5. Therefore, impairing the Mtr4/Trf interaction does not affect the steady-state level of CUTs.

Mutations in TRAMP complex or exosome subunits have also been shown to result in 3′ extended and polyadenylated snoRNA species. Thus, we next examined the effect of *trf5-Δ98–117* on two representative snoRNAs, the C/D box snoRNA snR128 and the H/ACA snoRNA snR33. As previously reported (18,19,29,43), 3′ extended species were detectable by northern blotting in *rrp6Δ* and *rrp44-exo^−^* strains. These species are detected as smears, instead of discreet products, indicative of their polyadenylation. We did not detect an increase in these species in the *rrp44-endo^−^* mutant. These polyadenylated snoRNA species have also previously been reported in *trf4* mutants, and consistent with this, we detected them in a *trf4Δ, trf5Δ* double mutant complemented with a wild-type *TRF5* plasmid. However, these species were less abundant in this *trf4Δ, trf5Δ* [*TRF5*] strain than in the exonuclease strains. Importantly, the *trf4Δ, trf5Δ* strain complemented with the *trf5-Δ98–117* plasmid reproducibly accumulated more 3′ extended snoRNAs than the same strain complemented with the wild-type *TRF5* plasmid. As previously described for exosome mutants (18,19), the steady-state level of mature snoRNAs was not altered in the *trf5-Δ98–117* strain (Figure 4C; 18,19). Thus, impairing the Mtr4/Trf interaction interferes with normal processing or degradation of these 3′ extended snoRNA species.

**Figure 5.**
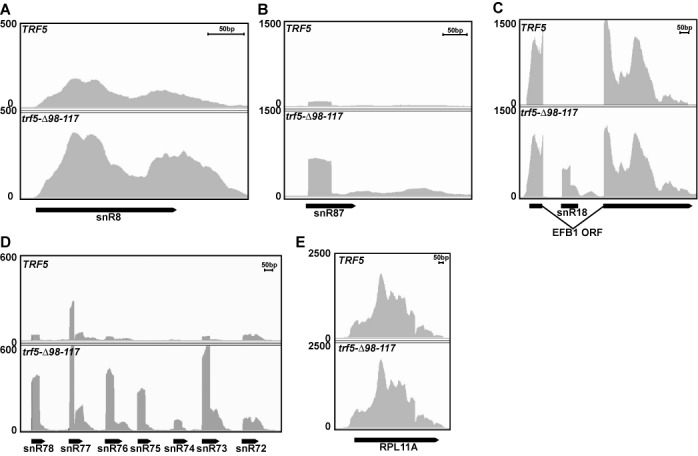
Disrupting stable TRAMP complex result in RNA processing defects of many snoRNAs. Poly(A)^+^ RNA from duplicate cultures of strains that express either wild-type Trf5 (top panels) or Trf5-Δ98–117 (bottom panels) were subjected to RNA seq. Results from only one culture of each are shown. (**A**) Results are shown for snR8, a monocistronically encoded H/ACA snoRNA that is not 5′ processed. (**B**) Results are shown for snR87, a monocistronically encoded C/D box snoRNA that is 5′ processed by Rnt1 and Rat1. (**C**) Results are shown for snR18, a C/D box snoRNA that is processed from the intron spliced out of the *EFB1* pre-mRNA. (**D**) Results are shown for snR72–78, a cluster of polycistronically encoded C/D box snoRNAs. These snoRNAs are separated from each other by Rnt1 and then further 5′- and 3′-end processed. (**E**) Results are shown for a representative mRNA (*RPL11A*). The peak covering the first 50 bp of snR87, 18 and 72–78 is explained by the 5′ monophosphate end of these snoRNAs being ligated to linkers during library preparation, combined with a 50-nt sequencing read length.

To study the effects of disrupting the Trf5-Mtr4 interaction more globally, we also analyzed duplicate samples of the *TRF5* and *trf5-Δ98–117* strains by transcriptome sequencing of poly(A)^+^ RNA. This analysis revealed that the set of most significantly affected genes (false discovery rate (FDR) = 0.01) included 71 that were overexpressed in *trf5-Δ98–117* (Table 1) and only five that were down-regulated. The set of 71 overexpressed genes was predominated by known TRAMP substrates, including 43 snoRNA genes. The snoRNAs detected as RNA-seq hits included both C/D box and H/ACA box snoRNAs. snoRNAs are processed from primary transcripts in a variety of ways. Figure 5 shows examples of monocistronically encoded snoRNAs that are either only 3′ processed (snR8; Figure 5A) or, in addition, are 5′ processed by Rnt1 and Rat1 (snR87; Figure 5B). Also shown is an example of a snoRNA that is processed from a spliced intron (snR18; Figure 5C). For this and other intron-encoded snoRNAs there was a clear increase in reads that mapped to the snoRNA and the part of the intron that is 3′ of the snoRNA, but there was no effect on the flanking protein-coding exons. Similarly, other mRNAs (such as RPL11A; Figure 5E) were not affected. Finally, Figure 5D shows an example of seven snoRNAs (snR72–78) that are transcribed as one polycistronic precursor. For each snoRNA there is a clear increase in the read density for both the snoRNA and the region just 3′ of the mature snoRNA. Strikingly, among the other genes that were detected as overexpressed are seven genes that are just 3′ of one of the overexpressed snoRNA genes (Table 1). The inclusion of these genes in the RNA-seq hits is likely due to the presence of 3′ extended polyadenylated snoRNAs. All of these changes were clearly reproducible in the duplicate transcriptome sequencing samples (e.g. Supplementary Figure S2). Thus, no particular kind of snoRNA appeared to be overrepresented among the RNA-seq hits. Although 23 other snoRNAs were not in the list of hits at the 0.01 FDR, most of these were significantly up-regulated at reduced stringency (*P*-values 0.004 to 0.05), and these snoRNAs also were not enriched for any particular kind of snoRNA. This set of snoRNAs detected as up-regulated at reduced stringency included snR33, which we had arbitrarily chosen to analyze by northern blot (Figure 4C; *P* < 0.004 for snR33). Finally, the RNA-seq hits included six other non-coding RNA loci that have previously been shown to be TRAMP and/or RNA exosome substrates (rRNA, U1 and U6 snRNA and the RNA subunits of the signal recognition particle, RNase P and RNase MRP; 18–20,33). These hits include RNAs transcribed by RNA polymerase I, II and III. Thus, poly(A)^+^ transcriptome sequencing data confirmed and extended our northern blot analysis. Overall, the RNA analyses of the *trf5-Δ98–117* strain indicate that many polyadenylated TRAMP substrates accumulate if Mtr4/Trf interaction is disrupted.

**Table 1.** Genes identified by poly(A)+ transcriptome sequencing as overexpressed in *trf5-*Δ*98–117*

Gene	Fold-change (log2)	*P*-value	FDR	Comments
RDN58	4.3	4.5E-126	1.6E-122	rRNA
YLR154W-E	4.1	6.7E-107	1.4E-103	overlaps with rRNA
SCR1	4.6	8.6E-056	1.3E-52	RNA component of SRP
snR128	2.3	2.7E-043	3.3E-40	C/D snoRNA
RDN5	2.5	1.0E-040	9.2E-38	rRNA
YLR154W-F	3.0	8.6E-037	6.5E-34	overlaps with rRNA
snR67	5.1	6.1E-028	4.1E-25	C/D snoRNA
RDN25	2.0	9.7E-028	5.4E-25	rRNA
snR40	4.2	7.9E-025	4.0E-22	C/D snoRNA
snR6	5.1	8.1E-024	3.8E-21	u6 snRNA
snR76	3.9	4.6E-020	2.0E-17	C/D snoRNA
RDN18	1.9	1.6E-019	6.3E-17	rRNA
snR87	2.9	1.2E-016	4.1E-14	C/D snoRNA
snR66	2.4	6.5E-016	2.1E-13	C/D snoRNA
snR18	4.1	6.6E-016	2.1E-13	C/D snoRNA
snR24	4.6	1.6E-012	4.9E-10	C/D snoRNA
snR34	1.5	8.7E-012	2.4E-09	H/ACA snoRNA
snR60	1.7	8.9E-012	2.4E-09	C/D snoRNA
EFM3	1.5	9.5E-012	2.4E-09	just 3′ of H/ACA snoRNA snR3
snR71	2.6	9.6E-012	2.4E-09	C/D snoRNA
snR17b	3.0	1.6E-011	3.9E-09	U3 snoRNA
YGR161C-D	1.2	5.9E-011	1.4E-08	TY1 transposon
snR3	1.5	2.7E-010	6.1E-08	H/ACA snoRNA
snR37	1.2	4.3E-010	9.2E-08	H/ACA snoRNA
snR77	1.4	1.2E-009	2.4E-07	C/D snoRNA
snR57	3.1	2.3E-009	4.7E-07	C/D snoRNA
snR56	1.1	6.3E-009	1.2E-06	C/D snoRNA
snR68	3.0	6.4E-009	1.2E-06	C/D snoRNA
snR52	3.6	7.1E-009	1.3E-06	C/D snoRNA
YJL047C-A	1.6	9.5E-009	1.7E-06	overlaps with C/D snoRNA snR60
snR73	4.2	1.1E-008	2.0E-06	C/D snoRNA
snR64	1.4	1.4E-008	2.3E-06	C/D snoRNA
snR61	2.5	1.8E-008	2.9E-06	C/D snoRNA
snR47	1.8	4.9E-008	7.6E-06	C/D snoRNA
snR45	1.7	5.6E-008	8.5E-06	C/D snoRNA
snR10	0.9	6.3E-008	9.2E-06	H/ACA snoRNA
RPR1	4.1	1.1E-007	1.5E-05	RNA component of RNase P
snR85	3.3	1.2E-007	1.7E-05	H/ACA snoRNA
YOL157C	2.0	1.3E-007	1.8E-05	
NME1	3.7	3.5E-007	4.7E-05	RNA component of RNase MRP;
snR13	1.3	4.1E-007	5.3E-05	C/D snoRNA
snR46	1.1	5.6E-007	7.2E-05	H/ACA snoRNA
snR82	1.3	6.0E-007	7.5E-05	H/ACA snoRNA
snR48	4.5	8.4E-007	1.0E-04	C/D snoRNA
snR32	0.9	1.9E-006	2.2E-04	H/ACA snoRNA
snR74	4.5	2.3E-006	2.7E-04	C/D snoRNA
snR35	1.2	2.8E-006	3.2E-04	H/ACA snoRNA
YOR040W	1.5	3.6E-006	4.1E-04	just 3′ of H/ACA snoRNA snr9
snR9	1.1	6.4E-006	7.0E-04	H/ACA snoRNA
LIN1	0.8	1.1E-005	1.2E-03	just 3′ of C/D snoRNA snr71
RDN37	1.9	1.4E-005	1.5E-03	rRNA
snR51	1.4	1.6E-005	1.7E-03	C/D snoRNA
YOR343W-A	3.1	1.8E-005	1.8E-03	TY2 transposon
PRP31	1.4	1.8E-005	1.8E-03	
snR54	3.1	2.5E-005	2.4E-03	C/D snoRNA
snR78	3.9	2.5E-005	2.4E-03	C/D snoRNA
BDF2	0.8	2.8E-005	2.6E-03	
snR42	1.0	2.8E-005	2.6E-03	H/ACA snoRNA
snR80	1.3	2.9E-005	2.7E-03	H/ACA snoRNA
MMS2	1.2	3.0E-005	2.7E-03	just 3′ of H/ACA snoRNA snr10
POP6	1.1	3.0E-005	2.7E-03	just 3′ of H/ACA snoRNA snr46
snR4	0.8	4.5E-005	3.9E-03	C/D snoRNA
snR19	2.8	4.6E-005	4.0E-03	U1 snRNA
YIL082W-A	8.5	5.7E-005	4.8E-03	TY3 transposon
snR69	3.1	5.8E-005	4.8E-03	C/D snoRNA
snR8	1.2	1.0E-004	0.008	H/ACA snoRNA
YPL222C-A	6.3	1.1E-004	0.008	dubious ORF
YGR161C-C	8.2	1.1E-004	0.008	TY1 transposon
YDR316W-A	8.2	1.1E-004	0.008	TY1 transposon
snR58	2.5	1.1E-004	0.008	C/D snoRNA
ODC2	0.8	1.2E-004	0.009	just 3′ of H/ACA snoRNA snr35

## DISCUSSION

Based on our new and previously published results we conclude that the TRAMP complex is composed of two well-folded catalytic cores that are brought together by two short peptide motifs. The majority of Mtr4 is well folded and forms an RNA-dependent ATPase core (32,37), while the polyadenylation catalytic core is assembled from well-folded domains of the Trf4/5 and Air1/2 subunits (44). In addition to these well-folded domains, each TRAMP subunit appears to have intrinsically disordered regions that function to mediate protein–protein interactions. Specifically, the N-termini of both Trf4/5 and Air1/2 contain small peptides that directly interact with Mtr4 (Figure 2; 38). Deletion of either one of these peptides impairs TRAMP complex formation such that Mtr4 is no longer immunoprecipitated with the Trf or Air subunit (Figure 3; 38,39). *In vitro*, each of these peptides establish a relatively low affinity interaction but combine for a high affinity interaction between the cores (38). We suspect that deleting one of the interaction sites eliminates formation of a stable TRAMP complex *in vivo*, since we and others have not been able to detect any TRAMP complex under such conditions (Figure 3; 38,39). However, we cannot exclude that a less stable or transient interaction mediated through the other peptide occurs in each of these experiments. In addition to these peptide motifs that mediate TRAMP complex assembly, the Mtr4 N-terminus contains a small motif that mediates interaction with the exosome cofactors Rrp6 and Rrp47 (45), the C-terminus of Trf4 contains a small motif that mediates interaction with Nrd1 (46), and the C-termini of Trf5, Air1, and Air2 contain short conserved motifs that may mediate interactions with additional factors (Supplementary Figure S1 and unpublished observations). Whether all of these interactions occur simultaneously or whether TRAMP dynamically associates with these factors remains to be determined.

While we were preparing this manuscript, Falk *et al.* independently identified similar Trf4 and Air2 regions that can directly interact with Mtr4 (38). Specifically, they showed that a complex of recombinant Trf4 and Air2 that contained either one of the peptide motifs interacted with Mtr4 with affinities very similar to those we determined. The agreement is remarkable considering the difference in methods (isothermal titration calorimetry/ITC versus fluorescence anisotropy), Mtr4 construct (Mtr4-ΔN80 and Mtr4-ΔN74) and Trf/Air binding partner (dimeric protein complexes versus isolated peptides). Furthermore, both the ITC and anisotropy measurements suggest that neither the N-terminus nor the arch domain of Mtr4 is required for these interactions. Overall, the *in vitro* data presented here and by Falk *et al.* suggest that the two peptides make equivalent contributions to TRAMP assembly.

The Falk *et al.* paper also presented a crystal structure of a Trf4-Air2 fusion peptide bound to Mtr4 (38). In this structure, Air2 residues 5–18 bind to the fist domain of Mtr4 (which is part of the arch domain). However, we detected no significant binding of Air2 residues 1–29 to the isolated fist domain in solution (Supplementary Figure S3), and both Falk *et al.* and our data show that Air2 binds with equal affinity to full-length and archless Mtr4 in solution, suggesting that the interactions with the fist do not make critical contributions to TRAMP assembly. Furthermore, the Falk *et al.* structure indicates that Air2 residues 1–29 make additional contacts with the helical bundle (also named ratchet) domain of Mtr4, but not the RecA domains, while our data indicate that the RecA domains of Mtr4 are sufficient for binding of Air2 residues 1–29 in solution (Supplementary Figure S3). Thus, the solution binding assays and crystal structure do not fully agree on the binding site on Mtr4 for Air2. One explanation for the discrepancy between the solution binding assays and crystal structure is that the interactions observed in the crystal structure may be influenced by the artificial tethering of the Air2 and Trf4 peptides that was needed to facilitate crystallization (38). Additional biochemical and structural characterization will be needed to fully define the Air2-Mtr4 interface. Importantly, our studies and the published crystal structure agree on the critical region in Trf4/5 that mediates Mtr4 interaction.

By deleting residues 98–117 of Trf5, we show that impairing TRAMP complex formation does not result in an obvious growth defect. Falk *et al.* showed that mutating residues in Mtr4-GFP residues critical for Trf4/5 interaction caused a slow growth phenotype (38). There are at least two explanations that can unite these observations. First, as discussed by Falk *et al.*, it is possible that the Mtr4 residues that interact with Trf4/5 are also important for some other aspect of Mtr4 function. Alternatively, it is possible that disrupting the Mtr4-Tr4/5 interaction is tolerated in a wild-type Mtr4 strain but not in an Mtr4-GFP strain. Consistent with this latter possibility is the observation that some other mutations in Mtr4 that have no growth phenotype in a wild-type Mtr4 strain, cause slow growth in the Mtr4-GFP strain (45). In either case, our results show that the Mtr4/Trf interaction is not required for viability. Both northern blot and transcriptome sequencing indicates that this strain has a defect in snoRNA processing and some other TRAMP functions. Most, if not all, snoRNAs accumulate as 3′ extended species in the poly(A)^+^ fraction. This suggests that these RNAs can still be polyadenylated by Trf5 but then fail to be degraded by the exosome. The simplest interpretation appears to be that stable TRAMP complex assembly is required to efficiently hand off substrates from the Trf subunit to the Mtr4 subunit and subsequently to the exosome. However, many alternative explanations cannot be excluded. For example, it has been shown that Mtr4 can modify the *in vitro* activity of Trf4 and *vice versa* (21,47), and thus impairing the interaction between Mtr4 and Trf4/5 may have effects beyond simply handing off substrates.

Our initial sequence analysis identified residues 98–117 to be conserved in orthologs of Trf4 and Trf5 from other yeast species, but conservation in other species was not readily detectable. However, it is notable that a multiple sequence alignment of PAPD5/hTFR4 orthologs from vertebrates also identifies a small conserved region in an otherwise poorly conserved N-terminus. The conserved sequence in vertebrates (EQxDFi/lP) is similar to the sequence conserved in yeast (d/nNxDFIxf/l). We therefore suspect that the interaction we describe between the yeast proteins is conserved in animals, even though standard sequence analysis tools fail to detect sequence conservation.

## SUPPLEMENTARY DATA

Supplementary Data are available at NAR Online.

SUPPLEMENTARY DATA
