# Supplementary material for: Interaction between the RNA-dependent ATPase and poly(A) polymerase subunits of the TRAMP complex is mediated by short peptides and important for snoRNA processing
Source: Nucleic Acids Res. 2015 Jan 14;43(3):1848–58. doi: 10.1093/nar/gkv005 (PMC4330371; doi:10.1093/nar/gkv005)
Supplement: SUPPLEMENTARY DATA [file supp_43_3_1848__index.html]

Interaction between the RNA-dependent ATPase and poly(A) polymerase subunits of the TRAMP complex is mediated by short peptides and important for snoRNA processing — SUPPLEMENTARY DATA 

# Interaction between the RNA-dependent ATPase and poly(A) polymerase subunits of the TRAMP complex is mediated by short peptides and important for snoRNA processing

## SUPPLEMENTARY DATA

**Files in this Data Supplement:**

- SUPPLEMENTARY DATA
