# Supplementary material for: Interaction between the RNA-dependent ATPase and poly(A) polymerase subunits of the TRAMP complex is mediated by short peptides and important for snoRNA processing
Source: Nucleic Acids Res. 2015 Jan 14;43(3):1848–58. doi: 10.1093/nar/gkv005 (PMC4330371; doi:10.1093/nar/gkv005)
Supplement: SUPPLEMENTARY DATA [file supp_gkv005_nar-03340-y-2014-File008.pdf]

Figure S1: Multiple sequence alignment of yeast Trf proteins generated with ClustalW and Boxshade. Species are Scer: *Saccharomyces cerevisiae*, Spar: *S. paradoxus*, Smik: *S. mikatae*, Sbay: *S. bayanus*, Skud: *S. kudriavzevii*, Scas: *S. castellii*, Sklu: *S. kluyveri,* Kpol: *Kluyveromyces polysporus*, Klac: *K. lactis*, Kthe: *K. thermotolerans*, Kwal: K. *waltii*, Cgla: *Candida glabrata*, Cgui: *C. guilliermondii*, Ctro: *C. tropicalis*, Calb: *C. albicans*, Cpar: C. *parapsilosis*, Clus: *C. lusitaniae,* Agos: *Ashbya gossypii*, Zrou: *Zygosaccharomyces rouxii*, Dhan: *Debaryomyces hansenii*, Lelo: *Lodderomyces elongisporus*, Ylip: *Yarrowia lipolytica,* Soct: *Schizosaccharomyces octosporus*, Spom: *Sch. pombe*, Sjap: *Sch. japonicus*

```
Trf5Scer     1 --------------------------MTRLKAKYSPTKGKRKE---------DKHTKRMR
Trf5Spar     1 --------------------------MTRPKAQYSPSKGKRKE---------NKHTKRMR
Trf5Smik     1 --------------------------MTRPKAQYSPSKGKSKE---------NKRIKRMR
Trf5Sbay     1 --------------------------MTRSKAQFSPSKGGKKE---------YKNPKRMR
Trf5Skud     1 --------------------------MKKAKVLFSPSKGGKKE---------NKNPNRMR
Trf5Cgla     1 --------------------------MRGDRSKRKVAKYQKKE---------SARVKRLR
Trf5Kpol     1 -----------------------------------MSKKRKLK---------IVKPKKLR
Trf5Scas     1 ----------------------MGAPVFLTHFIFMTRNKPKRTHSSPTKKVSKKTHRKIR
Trf4Scer     1 ------------------------MGAKSVTASSS-KKIKNRHNGKVKKSKKIKKVRKPQ
Trf4Spar     1 ------------------------MGAKSIAASST-KKIKKQHNGKVKKSKKIKKVRKSK
Trf4Smik     1 ------------------------MGSKSIAASSL-KKSKKQHSGKVKKSKKIKKSRKSK
Trf4Sbay     1 ------------------------MGVKSITTSSS-KKSKKQHNGKVKKNKKVKKVRKSK
Trf4Skud     1 ------------------------MGAKSIVASSSSKKSKKQHNGKMKKNKKIKKVRKSK
Trf4Kpol     1 ------------------------------------------------------------
Trf4Scas     1 ---------------------------MATKSTTSSIKQKPEKIKSKKIEISKGKGMKNS
Trf4Cgla     1 -----------------------------MSAAVDDVK----------------------
TrfKlac      1 ----------------------MSKRPSSGKGVK-PNRNRNKP---------VKQVKKMR
TrfAgos      1 -----MMTASVAGNRSGRSGNIARERSNRSRGAQKSDKERNLSKGSSRPKSNSSRVKKMR
TrfZrou      1 ----------------------------------------------------------MR
TrfKthe      1 ----------------------MGAKEASKNERAGPKSSSKSK----PLKNNQRRTKRLR
TrfKwal      1 ----------------------MGVKEGKKTERFGRKSSAKSK----PLKENQQKARRLR
TrfSklu      1 ----------------------MGRKKSQSPSLPQGSNSKNSKKLAYPLSAKKKKVKKLR
TrfDhan      1 ---------------------MMVSKRK--RESPPVKLKSKKQRKKTSKSGGIIPIENKY
TrfCgui      1 ----------------------MGNKRK--KEN------GKNSKPSTTKPG-FVESKNNY
TrfClus      1 ----------------MRKSTIMGQKRS--NDS-----QPAVTRKKARKTK--KKPQNTF
TrfCtro      1 ----------------------MSGNKR--KGGSINSTKPKKKKVKSPSVDVQLS--TNN
TrfCalb      1 ----------------------MGTKRK--RENLVGKPRSKRQKKDSLLLKVDNASFLEN
TrfCpar      1 ----------------------MGNKRQ--RERSDRKGDQKRQKKLNNVVAATTSPKHEN
TrfLelo      1 ----------------------MGSKRKHTKDGSMRDSSKKKRLSTAHGPPVEKRQRTSS
TrfYlip      1 MFSEVLSPMAKRTTKLPPATVATKADLRNRPIAQPVSRRRGQQATPNNANNNSNNNSAED
TrfSoct      1 --------------------MAKKSTFSKANKANKIKKKSKFDEREPLPRRIFKKDDRNR
Cid14Spom    1 --------------------MGKKSVSFNRNNY----KKRKNERTEPLPRRIFKNDKPSK
TrfSjap      1 --------------------MSKKKSKDSNSKKNR-PNSSKGESKGSLARRIFKRGSKSK
```

```
Trf5Scer    26 KSSFTRTQKMLEVFNDNRSHFNKYESLAIDVEDDDTFGNLVLMEND--------------
Trf5Spar    26 KSSFTRTQKMLEVFNDNRSHFNKYESLAIDVDDDGTFGNFVLMEND--------------
Trf5Smik    26 KSSFTRTQKMFEVFNDNRSHFNKYESLAIDVDDDGTLGNFVLMKND--------------
Trf5Sbay    26 KSSFTKTQKMFEVFNENRSHFNKYESLAMDVDDDGTFGNFVLMENS--------------
Trf5Skud    26 KTSFTRTQKMFEVFNDNRSHFNKYESLAIDIDDDGTFGNFVLMENN--------------
Trf5Cgla    26 RSSFNALQRNIEVFNDNKEFLNKYENLELDLSDPENRTHLLSEYNDDLTQ----------
Trf5Kpol    17 KSSINKIRKNFNLFNEIDDIDNYDKLIDMEKSN-NNRFLVLQD-----------------
Trf5Scas    39 KSSISRIEKQFKNFDNDIDQYNKYENLALDVSDTEELLQQDRKGHHFSFIEEDVREEVPG
Trf4Scer    36 KSISLNDENEVEILPSRNEQETNKLPKDHVTADGILVLEHKSDDDEGFDVYDG-------
Trf4Spar    36 KPKSLSDENGVEIVSSRNDQETNTSLKDHVTADGILVLEHKSDDDEGFDVYDG-------
Trf4Smik    36 KSKSLSDENGAEVTSSRSEQDNNKSPDDHVTEDGILVLEHKSDDDEGFDDYDG-------
Trf4Sbay    36 KPRSTSDDNEAESSVKDNEEESKKASGDHVTADGILVLENKSDDDEGFDDYDG-------
Trf4Skud    37 KDTSTSDENEAGNLPKLNDQEINKNPDDHVTSDGILVLEKKSDDDEGFDDYDG-------
Trf4Kpol     1 --MSTTEKN----------------------KDIVIINDNVSEENDDYDDYDG-------
Trf4Scas    34 SKSKNSKHGKKSMRRSKNKNQNNKPPTTVRSTDGITFLESSS-ESEGYDDYDG-------
Trf4Cgla    10 ---------------------------TVEDVADVIAVESS--DSQGFDDYDG-------
TrfKlac     29 KSSLSRAQKNFSVFQQ-QSHENKYESLQSIRDDGEDAGDEVVFLHDEGKD-----LNSDD
TrfAgos     56 KSSLSKVQKNFAVFNDSAEHENKYETLAVAEADEK----EVILLDEE-------------
TrfZrou      3 KSSLSKAQRNFQVFNR-NDHVNQYENLPESAVEDK-------------------------
TrfKthe     35 KSSLSKAQRNFAVFNKDGAHINKYENLELDNIESEG-DDGSER-----------------
TrfKwal     35 KSSLSKAQRSFAVFNKDNSHLNKYENLKMEVIESEG-DNANES-----------------
TrfSklu     39 KSSLSKAQKNFAVFNKMDEHINKYENLPVDISDEEHNDNHSDKDGNPSFTHDGMILIDEY
TrfDhan     38 EALPSSDNESDSS-----LKFDFKSNSREP---EMVFVESDDEEEGVVIVSDDDKPE---
TrfCgui     30 DVLSS---DSDSS-----AIFDH--NRKDE---NLVLVDSSDDD--IVVESDSENDEN--
TrfClus     36 ETLPP---EPQDD-----IVLVESGDESHS---DVVVFSDREEE---TQETETKEAK---
TrfCtro     35 NFVALQDHVDEDS--DIEVIYEYKKPQQE-----LVLLDSEEDE------DEDAENEV--
TrfCalb     37 KFETLQNHLDEES--DSELIYDYDRKKSEELTDDLVLIDSEEES------NDSSD-----
TrfCpar     37 NFIKLP-HSDDDS--DSDYIFDYSRKSQSP-EKELILVDSDAEK------NSGDS-----
TrfLelo     39 NTYESLYEIDGESSSDSEHIFNYDKDTSPEDKNVINLIDSDEDD------KEEKGEEE--
TrfYlip     61 KSNNTNDTKDSKDGKEKASKKKRKKNKKKSQSGPISPQDRRNSGNGYNVLVDENDDKSGD
TrfSoct     41 -RFKKNDGKRKSKYPDELEVNNNFSLLE-QEQ-----GENRKESDG-TQYSQQKNDIDNP
Cid14Spom   37 FKSKRKEKDKNSDAYDEMLLNNNFTLLD-QEEPMVEIGSKKSRNDNDSEGIRDKGGVEIS
TrfSjap     40 -RKSSESKKHKRTVYDDLSGENNFSVLDNQDPDVIEKSETEPESSNVSSSTETAVSSDVS


Trf5Scer    72 -----------KSDVDIPVIEEVTSSEDEQRAESS-------------------------
Trf5Spar    72 -----------KGDVDIPVIEENTSSDDEKRIESR-------------------------
Trf5Smik    72 -----------KVDVEIPVIEEGTSSDEEGQTESR-------------------------
Trf5Sbay    72 -----------EGDIDIPVIEEETSGDDETINESL-------------------------
Trf5Skud    72 -----------KGDIDIPVIEEDTSDDDKKMKGSR-------------------------
Trf5Cgla    76 -SDGESKADNHNDSYDTIPVIDIDSGDEGIVSDIK-------------------------
Trf5Kpol    59 -TDG-------IDKIDEIPIVDIESDTS--NDDID-------------------------
Trf5Scas    99 ----------TSDVPIGIFDSENQSSTETKIDACPT------------------------
Trf4Scer    89 ----------HFDNPTDIPSTT-EESKTPSLAVHG-------------------------
Trf4Spar    89 ----------HFDNPTDIPSTT-EESRTPSLAVHG-------------------------
Trf4Smik    89 ----------HFDNPTDIPSTT-EGSKTPLLGVHG-------------------------
Trf4Sbay    89 ----------HFDNPTDVPSTA-EGSKTPLLVVHG-------------------------
Trf4Skud    90 ----------HFDNPTDVPSTT-EENKTPLLTVHG-------------------------
Trf4Kpol    30 ----------HIGNPLTISDSS-AESNNGDNKVPK-------------------------
Trf4Scas    86 ----------HFDNPTESEDKDLKMAKVAVEKVPQ-------------------------
Trf4Cgla    34 ----------HNDSPKE---------------MPI-------------------------
TrfKlac     83 IDEVSVSENDLDLEDEEDEEEGEEEEPVGTADGI--------------------------
TrfAgos     99 --------EDTAMTTETKGVHAEETANVTGAEGVN-------------------------
TrfZrou     37 ------------------------------------------------------------
TrfKthe     77 ----------TQESSEAPTTEATPESEEPQKEQA--------------------------
TrfKwal     77 ----------TKIS-EDEVVNISSGSDENRVSQG--------------------------
TrfSklu     99 DDQDIPNDSIIKDVKEVDVVDLDSEEENMMLNNNN-------------------------
TrfDhan     87 ------------------------DSEEPASKKQ--------------------------
TrfCgui     73 -----------NSETDTKKRKVDSDNHQPASKKR--------------------------
TrfClus     79 ------------------------AIDSIPETAD--------------------------
TrfCtro     80 -----------ETKKSVDQSNPKKRKFDEVNEADDD------------------------
TrfCalb     84 ---------------GFDDINSESEKKQVLVGND--------------------------
TrfCpar     82 -----------------DSNESIKDSTESSHENQ--------------------------
TrfLelo     91 -----------EEEEEEDDDENGNSSGENQIEIDGDKVKDKEISPSDQEKLIESTSTSST
TrfYlip    121 EGERSSKKRRTAGSAASTTASSDMDSENERSVSTAPTSAPAPKPPAD-------------
TrfSoct     93 SK-PSLNQSGRVDPLETLEKLDLPEEAIKKGAPSILVGLKKLDDKSE-------------
Cid14Spom   96 NKNDPYIQFGKADPLEPLEKPDLPEEAIKRGEPTILLGIPKREGR---------------
TrfSjap     99 FAEPRAQTHGKVDPLDVLDKPDLPDEAIKRGEPSILLGVPKVKKR---------------
```

```
Trf5Scer    96 ---------KRNNSLEDNQDFIAFSDSSEDETEQIKEDDDERSSFLLTDEHEVSKLTS--
Trf5Spar    96 ---------KRGHSLEDNHDFIAFSDSSEEEKEQIKEDEDERSSFLLADQYKTSTFSS--
Trf5Smik    96 ---------KGDHSLEDNHDFIAFSDTSEDEKEQKRGEDEEKCDFLSLNQYEISTFGS--
Trf5Sbay    96 ---------KNGNSLEDNHDFIAFSESSGDEE-EEERVTEEKSVLLVHDQYEISTVTS--
Trf5Skud    96 ---------EKRNSLEDNQDFIPFFDSSEDEQGEEEGNSEGVSGFLMPDQYEITKNNT--
Trf5Cgla   110 ---------ISGGKLEDNDDFIAFSSSSEDENSGA-ENNESEDYLSSGNTSSD-------
Trf5Kpol    84 ---------NLEDSLKDNNDFIAVSSTSEED-----YDDISEDEDTDGNESS--------
Trf5Scas   125 --------LLQLNKLANNDDFIPFSASSEDEDEDDEGPLNPTINDYGETLANR-------
Trf4Scer   113 ---------DEKD-LANNDDFISLSASSEDEQAEQ-EEEREKQELEIKKEKQK-------
Trf4Spar   113 ---------NEKD-LANNDDFISLSASSEDEQAEQ-EEERQKEELEVKKKKQK-------
Trf4Smik   113 ---------DEKD-LANNDDFISLSASSEDEHAEQ-EEERQKE------EKQK-------
Trf4Sbay   113 ---------NEND-LANNDDYIALSASSEDEDAKQKEEEKQKKEEEILKRKQK-------
Trf4Skud   114 ---------SERD-LANNDDFISLSASSEDEDAKKEEEERQKKEAELLRIRQK-------
Trf4Kpol    54 ---------LRSD-IDTNNDFIAFSDSSEDEKENDDINEKESK------DEDA-------
Trf4Scas   111 ---------LQNNKLEDNQDFIAFSASSSDEKDIQEKQALEVDEQ--VTKEPT-------
Trf4Cgla    44 ---------IDNNQIDENDDFIAFSESSGGE---DGDEAEEEQPQ--AEEEE--------
TrfKlac    117 ---------IGNNSLDANEDFIAFDLSEPEEDEHENGFEEHSEPETAGKEEIE-------
TrfAgos    126 ---------AAANALDDNQDFIGFSDSEEEVASGE----ENGDADYA-VEDAE-------
TrfZrou     37 -----------NGSLDGNDDFIAFDDSSSEDEGGK----EEQE-----TESVE-------
TrfKthe    101 ----------QDNALDDNQDYISLSMSSEEEQESE---EEAPDEAGGEHPGGD-------
TrfKwal    100 ----------EENALEDNQDYISLSMSSDAADVSS---EEEGHSDNVGSDDDY-------
TrfSklu    134 ---------NQQNALDNNEDFIPFAVSSEEEDEEK---DEHGTYDYDDYDDEYPSKEEE-
TrfDhan     97 --------KFVNNELAKNQDFIEFGFSSSEEDAPERNDYDDYSDDGVLSDDESGTI----
TrfCgui     96 --------KMEYNELSKNQDFIEFGFSSSEE---EANKYDDYSDDGIISDDQSGNR----
TrfClus     89 --------AAPPNDLTNNNDYIRLGLSSSED------EAEDLSDDGVLSDDESGSR----
TrfCtro    105 -------ITTETNELAKNEDFIGFGFSSTDEEE----SEGEE---------SEND-----
TrfCalb    103 --------KTPENELANNEDFIGFGFSSEDDSEEISNSDNDL---------SNGDY----
TrfCpar     99 ----------ESNELTTNQDFIQFGFSSSDEDDE--HDEEHD---------SQNTN----
TrfLelo    140 TSSTSSTNLPEANDLAKNDDFIEFGFLSSDEDEDEDRDDGNGGINSVAEEIDDTSR----
TrfYlip    168 --SPPPPPPVDPSEWNMNEDFVGFDFSDGEDEEDEEPEEPVRSRKRTIEEVDDASS----
TrfSoct    139 -----KPKPEPKNAVDENADYIGFDWNSDEDVNDTSKDETND------SGPNNIPGFMQN
Cid14Spom  141 -----KTNPVHDKAVENNSDFIKFDWNSDEDEDSVSNDKSKNNESLKKSSKNEIPGFMRQ
TrfSjap    144 -------KSIGDASVEGNADFIKFDFSTDDEEEDKEDETSRR-----EEKKSAIPAFMQK


Trf5Scer   145 -----------------QQSLNTESACNVEYPWIRNHCHSKQRRIADWLTSEIKDFVHYISP
Trf5Spar   145 -----------------QHPQNTDSACNTEYPWIRNHCHSRQRRIADWLTSEIKDFVHYISP
Trf5Smik   145 -----------------QYHQDTESICNAEYPWIRDHCHSSQRRIADWLTLEIKDFVRYISP
Trf5Sbay   144 -----------------ISHENGDSVCNTEYPWIKDHGHSRQRGIADWLTLEIKDFVHYISP
Trf5Skud   145 -----------------QSHENTHSTSNTEYPWIRNHNHSRQRKIAEWLTSEIKDFVHYISP
Trf5Cgla   153 --------------DELAGRRNAERELNADFPWLLNHDHSKQKEISDWLTAEIRDFVAYISP
Trf5Kpol   122 --------------------HADERTLNTEFPWLLNHDHSKQKEIIDWLTLEIKDFVAYISP
Trf5Scas   170 --------------------YEYTSSVNVHHPWILNHDHTEQRSISAWLTTEIIDFVSYISP
Trf4Scer   155 ------------------------EILNTDYPWILNHDHSKQKEISDWLTFEIKDFVAYISP
Trf4Spar   155 ------------------------EILNTDYPWILNHDHSKQKEISDWLTFEIKDFVAYISP
Trf4Smik   149 ------------------------EILNTDYPWIVNHDHSRQKEISDWLTFEIKDFVAYISP
Trf4Sbay   156 ------------------------EILNTDYPWILNHDHSKQTEISDWLTFEIKDFVAYISP
Trf4Skud   157 ------------------------EILNTDYPWILNHDHSRQKEISDWLTFEIKDFVAYISP
Trf4Kpol    91 ------------------------NILNMDFPWTINRDHSKQKEIADWLTLEIRDFVSYISP
Trf4Scas   153 ------------------------KILKSDYPWIINHDHSKQKEISDWLTLEIADFVSYISP
Trf4Cgla    82 ---------------------------YTDYPWTIDRDYSKEKELADWLNYEILDFVAYISP
TrfKlac    161 -----------------EGEGDKLR-HNADFPWILNHDHSKQREIADWLTLEIKDFVSYISP
TrfAgos    165 -----------------DESSEPLP-TNADYPWIQNHDHSRQREIADWLTLEIKDFVSYISP
TrfZrou     70 -----------------ESGEQVLPTVNTDYPWILNHDHSKQREIADWLTLEIRDFVAYISP
TrfKthe    141 --------------------AAQPRTLNTDYPWILNHDHSKQREIADWLTLEIKDFVAYISP
TrfKwal    140 --------------------EGVPKTLNTDFPWILNHDHSKQREIADWLTLEIKDFVAYISP
TrfSklu    181 --------------EQNAANIAAKRTLNTDYPWVLSHDHSKQKEIADWLTLEIKDFVSYISP
TrfDhan    145 ---------------------HTQTTTKSLYPWIKDHDHSKQKEIADWFTMEIKDFVNYISP
TrfCgui    141 ---------------------HIAKEPSSLYPWIKNQDHSKQQEIADWLTLEIKDFVNYISP
TrfClus    131 ---------------------HHSQSATSPYPWVKGHDHSKQREIADWLTMEIKDFVNYISP
TrfCtro    140 ---------------------DHSGAPNSQYPWIKDHDHSTQKEIADWLTMEIRDFVNYISP
TrfCalb    142 ---------------------AHGETPNSSYPWVKDHDHSKQKEIADWLTLEMKDFVNYISP
TrfCpar    134 ---------------------ELSAEANSSYPWIKDHDHSTQKEIADWLTMEMKDFVRYISP
TrfLelo    196 ---------------------PEDLHTNSQYPWVKNHDHSRQKEIADWLTMEMKDFVNYISP
TrfYlip    222 --------------------GPASKKQDLEAPWIKSNYHQYDR-VSDWLQEEVMDFVKYISP
TrfSoct    188 NGRFFHEANKQNEQSSRKRKRMAYEIDPSSCPW--HRPYKADIEVSRLLHQDITQFVDYITP
Cid14Spom  196 RGRFFHEANEKSD-SNRKRKRQAYELDSQSCPW--HRQYKVEREVSRIFHQDILHFIDYITP
TrfSjap    192 MGRFFRDK-----VSNRKRKRNAMDIEP-ICAW--HKPYPEEPEVARLLHQDILNFINYLEP
```

```
Trf5Scer    190 SKNEIKCRNRTIDKLRRAVKELWSDADLHVFGSFATDLYLPGSDIDCVVNS-RNRDKEDR
Trf5Spar    190 SKSEIKCRNRTIDKLRQAVKELWSDADLHVFGSFATDLYLPGSDIDCVINS-RNRDKEDR
Trf5Smik    190 SKSEIKCRNRTIDKLRQAVKELWRDADLHVFGSFATDLYLPGSDIDCVINS-RNRDKEDR
Trf5Sbay    189 SKAEIKCRNRTIDKLRQAVKELWSDADLHVFGSFATDLYLPGSDIDCVINS-RNRDKEDR
Trf5Skud    190 SKSEIKCRNRTIDKLRQAVKQLWSDADLHVFGSFATDLYLPGSDIDCVINS-RHHDKEDR
Trf5Cgla    201 SREEIETRNKTIAKIRRSVKRLWTDADLQVFGSYATDMYLPGSDIDCVVNS-KSGDKENR
Trf5Kpol    164 SKEEIEIRNVTISKIRNSLKELWSDSDLHVFGSYATDLYLPSSDIDCVVNS-ENGDKENR
Trf5Scas    212 SKDEIHTRNRTLARLRKAVSEQWKDASLHVFGSYATDLYLPGSDIDCAVIS-RNRDKDRR
Trf4Scer    193 SREEIEIRNQTISTIREAVKQLWPDADLHVFGSYSTDLYLPGSDIDCVVTS-ELGGKESR
Trf4Spar    193 SREEIEIRNQTISSIREAVKQLWPDADLHVFGSYSTDLYLPGSDIDCVVTS-ELGGKESR
Trf4Smik    187 SREEIEIRNQTISTIREAVRQLWPDADLHVFGSYSTDLYLPGSDIDCVVNS-ELGGKESR
Trf4Sbay    194 SREEIEVRNQTISTIREAVKQLWSDADLHVFGSYSTDLYLPGSDIDCVVNS-ELGGKESR
Trf4Skud    195 SREEIEIRNQTISTIREALKQLWPDADLHVFGSYSTDLYLPGSDIDCVVNS-ELGGKESR
Trf4Kpol    129 NRKEIELRNQTIGKLRDAVQHHWPDANLHVFGSYATDLYLPGSDIDCVVNS-KAGDKQSR
Trf4Scas    191 SREEIESRNQTISKVRNAVKQLWPDADLHVFGSYATDLYLPGSDIDCVINS-KAGDKENR
Trf4Cgla    117 SKEEIETRNRTIGSIRSAVKELWPDADLHVFGSYATDLYLPGSDIDCVVNS-KQGDKQSR
TrfKlac     205 NRQEIEQRNQAIAKLKEAVVELWPDSSLNCFGSYATDLYLPGSDIDCVVRS-ASGDKENR
TrfAgos     209 NKTEIQLRNDALKRIRDAVQDFWPDANLHCFGSYATDLYLPGSDIDCVVNS-KSGDKDNK
TrfZrou     115 SRQEIELRNKTIRTLRHAVRKLWPGADLQVFGSYATDLYLPGSDIDCVINS-KTGDKENR
TrfKthe     183 SQEEIRVRNSTISKIRNAVKDLWPDADLHVFGSFATDLYLPGSDIDCVINS-SSGDKENR
TrfKwal     182 SQEEIRVRNSTISTIRAAVRELWPDADLLVFGSFATDLYLPGSDIDCVINS-TKGDKQNR
TrfSklu     229 SREEIETRNFTISKVRDAVQQLWSDADLHVFGSFATDLYLPGSDIDCVVNS-RSGDKENR
TrfDhan     186 SEAEIMTRNRVVNQLKQQIGQFWPATELHVFGSCATDLYLPGSDIDMVVVS-ETGDYEHR
TrfCgui     182 SKLEITTRNNVIGRLKSTITKFWPDTEVHVFGSSATDLYLPGSDIDMVVIS-RDGDREQR
TrfClus     172 SKEEIVVRNTVIRRLKRRIAEFWPQTQAHVFGSCATDLYLPGSDIDMVVIS-TTGDYEQR
TrfCtro     181 SSDEIITRNKVIAALKKSISDFWPGTTVHVFGSCATDLYLPGSDIDMVVVS-DTGSYENA
TrfCalb     183 SSEEIVTRNNVISTLKKEIGKFWPGTETHVFGSCATDLYLPGSDIDMVVVS-ETGDYENR
TrfCpar     175 SKAEIITRNNVINTLKKEISSFWPGTEAHVFGSCATDLYLPGSDIDMVVIS-STGDYENR
TrfLelo     237 SSEEIVIRNKVVNTLKTQIALFWPGTEAHVFGSSATDLYLPGSDIDMVVLS-DTGDYENR
TrfYlip     263 TQSEIKARQDLVERVRGAVNGLWGDAKVHVLGSFTTNMHLPQSDIDLVVCS-PHGHYGER
TrfSoct     248 TPEEHAVRKSLISRINNAVVKKWPDVSVYVFGSFETRLYLPTSDLDMVIMSSDSHYRGSK
Cid14Spom   255 TPEEHAVRKTLVSRINQAVLQKWPDVSLYVFGSFETKLYLPTSDLDLVIISPEHHYRGTK
TrfSjap     246 TPQEHAVRKSLITKLDRAIRAKWPEVTVYVFGSFETRLYLPTSDIDMVVMSSDTVHRGTK


Trf5Scer    249 NYIYELARHLKNKGLAIRMEVIVKTRVPIIKFIEPQSQLHIDVSFERTNGLEAAKLIREW
Trf5Spar    249 NYIYELARYLKNKGLAIRMEVIVKTRVPIIKFIEPQSQLHIDVSFERTNGLEAAKLIREW
Trf5Smik    249 NYIYELARYLKTKGLAIRMEVIVKTRVPIIKFIEPQSQLHIDVSFERTNGLEAAKLIREW
Trf5Sbay    248 NYIYELARHLKNQGLAIRMEVIVRTRVPIIKFIEPQSQLHIDVSFERTNGLEAAKLIREW
Trf5Skud    249 NYIYELARYLKNEGLAIRMEVIVRTRVPIIKFIEPLSQLHIDVSFERTNGLEAARLIREW
Trf5Cgla    260 QYLYELARHLKNDGLATRVEVIAKSRVPIIKFVEPESDIHIDVSFERSNGLEAAKLIREW
Trf5Kpol    223 NDLYSLATHFKRNGLAIQVEVIAKARVPIIKFVEPNSKLHIDISFERLNGLEVAKIIREW
Trf5Scas    271 QCLYDLAKSLKQKGLATHLEVIAKARVPIIKFVEPRSKIHIDVSFEKTNGAEAAKLIREW
Trf4Scer    252 NNLYSLASHLKKKNLATEVEVVAKARVPIIKFVEPHSGIHIDVSFERTNGIEAAKLIREW
Trf4Spar    252 NNLYSLASHLKKKNLATEVEVVAKARVPIIKFVEPHSGIHIDVSFERTNGLEAAKLIREW
Trf4Smik    246 TNLYSLASHLKKNNLATEIEVVAKARVPIIKFVEPHSGIHIDVSFERTNGLEAAKLIREW
Trf4Sbay    253 NNLYSLASHLKKNNLATEIEVVAKARVPIIKFVEPHSGIHIDVSFERTNGLEAAKLIREW
Trf4Skud    254 NNLYSLASHLKKNNLATEIEVVAKARVPIIKFVEPHSRIHIDVSFERTNGLEAAKLIREW
Trf4Kpol    188 NCLYSLASHLKKEGLAEDIEIIAKARVPIIKFVEPLSKIHVDVSFERTNGLEAAKLIRGW
Trf4Scas    250 NSLYSLASFLKQQGLATQIEVIAKTRVPIIKFVEPESNIHIDVSFERTNGLEAAKLIREW
Trf4Cgla    176 NNLYKLANFLKKKEIATEIEVVAKARVPIIKFVEVESRTHMDISFERLNGLEAAKLIRDW
TrfKlac     264 NALYSLASFLKRKQLATQVEVIAKARVPIIKFVEPESKIHIDVSFERTNGLEAARVIRGW
TrfAgos     268 NALYSLASYLKRNGLATQVSVIAKARVPIIKFVEPASQIHIDLSFERTNGVEAAKIIRGW
TrfZrou     174 SSLYELAHFLKNRKLATQVEVIAKARVPIIKFVEPTSQIHVDVSFERTNGLEAAKLIRSW
TrfKthe     242 NCLYSLASFLKRRKLATQVEVISKARVPIIKFVEPISQIHIDISFERVNGLEAARVIRGW
TrfKwal     241 NCLYSLASFLKRKNLATQVEVISKARVPIIKFVEPSSQIHIDISFERVNGVEAARIIRGW
TrfSklu     288 NALYSLASFLKRRKLATQVEVISRARVPIIKFVEPESGIHIDVSFERTNGLEAARIIRNW
TrfDhan     245 SRLYQLSSFLRNKKLAKNIEVIAKAKVPIIKFVDPTSNIHIDISFERTNGIDAAKKIRRW
TrfCgui     241 SRLYQLSTHLRSKKLAKNIEVIAKAKVPIVKFVDPDSNIHIDVSFERSNGIDAAIKIREW
TrfClus     231 GKLYQLSSFLRTNKLAKNIEVIATAKVPIIKFVDPQYNIHVDISFERTNGLDAARRIRKW
TrfCtro     240 SRLYQLSTFLRTNKLATEVEVIAHAKVPIIKFVDPKSRLHIDVSFERTNGIDAAKRIRRW
TrfCalb     242 SRLYQLSTFLRTKKLAKNVEVIASAKVPIIKFVDPVSELHIDVSFERTNGLDAAKRIRRW
TrfCpar     234 SRLYQLSSFLRVKNLAKNVEVIANAKVPIIKFVDPDSNLPVDISFERTNGLDAARRIRKW
TrfLelo     296 SRLYQLSSFLKAKKLATNVEVIASAKVPIIKFVDPDSNIHVDISFERKNGLDAARRIRRW
TrfYlip     322 ACIYQLSSVIRSRMKVAELQTITKARVPIIKFIDSRTGVHVDISFEKDGGIKTASTITKW
TrfSoct     308 KDMFVLAHHLRKSKVATDIQVITTANVPIIKFVDPITKIHVDVSFNQAGGLKTCLVVNGF
Cid14Spom   315 KDMFVLAHHLKKLKLASEVQVITTANVPIIKFVDPLTKVHVDISFNQPGGLKTCLVVNGF
TrfSjap     306 KHMYSLARHLKNCKLATEIQVITTANVPIIKFVDPFTRIHVDVSFNQPGGLKTCLVVNGF
```

```
Trf5Scer   309 LRDSPGLRELVLIIKQFLHSRRLNNVHTGGLGGFTVICLVYSFLNMHPRIKSNDIDVLDN
Trf5Spar   309 LRDSPGLRELVLIIKQFLHSRRLNNVHTGGLGGFTVICLVYSFLNMHPRIKSNDIDAPDN
Trf5Smik   309 LRDSQGLRELVLIIKQFLHSRRLNNVHTGGLGGFTVICLVYSFLNMHPRIKSNDIDALDN
Trf5Sbay   308 LSDSPGLRELVLVIKQFLHSRRLNNVHTGGLGGFTVICLVYSFLNMHPRIKSNDIDAPDN
Trf5Skud   309 LRDSPGLRELVLVIKQFLHSRRLNNVHTGGLGGFTVICLVYSFLNMHPRIKSNDIDTPDN
Trf5Cgla   320 IGDTPGLRELTLVVKQFLHARRLNDVHTGGLGGFSIICLVFSFLRLHPRIITGDIDPLDN
Trf5Kpol   283 LDDTPGLRELVLIVKQFLAARRLNNVHTGGLGGLSIICLVYSFLKLHPRIITNDIDPIDN
Trf5Scas   331 IKDTPGLRELVLVLKQFLAVKKLNEVVNGGLGGFSIICLVYAFLRMHPRIKAGEIDPMKN
Trf4Scer   312 LDDTPGLRELVLIVKQFLHARRLNNVHTGGLGGFSIICLVFSFLHMHPRIITNEIDPKDN
Trf4Spar   312 LNDTPGLRELVLIVKQFLHARRLNNVHTGGLGGFSIICLVFSFLHMHPRIITNEIDPKDN
Trf4Smik   306 LNDTPGLRELVLIVKQFLHARRLNNVHTGGLGGFSIICLVFSFLHMHPRIITNEIDPKDN
Trf4Sbay   313 LNDTPGLRELVLIVKQFLHSRRLNNVHTGGLGGFSIICLVFSFLHMHPRIITREIDPKDN
Trf4Skud   314 LNDTPGLRELVLIVKQFLHARRLNNVHTGGLGGFSIICLVFSFLHMHPRIITKEIDSKDN
Trf4Kpol   248 LDSTNGLRELVLIVKQFLQARRLNKVHTGGLGGFSIICLVYSFLHLHPRILANEINPIEN
Trf4Scas   310 LQDTPGLRELVLIIKQFLHSRRLNNVHTGGLGGFSIICIVFSFLQMHPRIITNEIDPMEN
Trf4Cgla   236 LASTPGLRELVLVVKQFLHSRRLNNVHSGGLGGFSIICLVYSFLRMHPRIITAEIDPLEN
TrfKlac    324 LEEQPGLRELVLIVKQFLHARRLNNVHTGGLGGYSIICLVYTFLKLHPRVLTGDIDPLEN
TrfAgos    328 LHDTPGLRELVLIVKQFLHARRLNDVHIGGLGGFSIICLAYSFLKLHPRIICRDIEPLQN
TrfZrou    234 LQQTPGLRELVLIVKQFLHARRLNNVHTGGLGGFSIICLVYAFLNLHPRIVTGEIDARYN
TrfKthe    302 LKETPGLRELVLIVKQFLAARRLNMVHTGGLGGFSIICLVYAFLQLHPRVISSDIDSMEN
TrfKwal    301 LNDTPGLRELVLIVKQFLAARRLNIVHTGGLGGFSIICLVYSFLQLHPRVISSDINSMEN
TrfSklu    348 LDETPGLRELVLIVKQFLSARRLNNVHTGGLGGFSIICLVYSFLQLHPRILTNDINSLEN
TrfDhan    305 LSSTPGLRELVLIVKQFLRSRKLNNVHVGGLGGYSTIILCYHFLKLHPRLSTKNMAVLDN
TrfCgui    301 LASTPGLRELVLVVKQFLRSRRLNNVHVGGLGGYSTIILCYHFLKLHPRVATENMSILDN
TrfClus    291 LDSMPGLRELVLIVKQFLRSRRLNNVHVGGLGGYATIILMYHFLRLHPRVSTGNISVMEN
TrfCtro    300 LVSTPGLRELVLVVKQFLRTRRLNNVHVGGLGGYATIIMCYHFLRLHPKITTDAMSALDN
TrfCalb    302 LISTPGLRELVLVIKQFLRSRRLNNVHVGGLGGYATIIMCYHFLRLHPKLSTSSMDALDN
TrfCpar    294 LLATPGLRELVLVVKQFLRSRKLNNVHVGGLGGYATIIMCYHFMQLHPKISTNTMDAPDN
TrfLelo    356 LASTPGLRELVLVVKQFLRSRKLNNVHVGGLGGYATIIICYHFLRLHPKLSTESMSSLDN
TrfYlip    382 SKSMPALRPLVLIVKQFLSSRRLNEVFTGGLGGFSVICMVYSFLIQHPRISTGDISPMDN
TrfSoct    368 LKKYPALRPLVIIIKHYLNMRALNEVFLGGLSSYAIVCLVVSFLQLHPRLSTGSIREEDN
Cid14Spom  375 MKKYPALRPLVIIIKHFLNMRALNEVFLGGLSSYAIVCLVVSFLQLHPRLSTGSMREEDN
TrfSjap    366 LKKFPAVRPLTMLVKHFLNMRALNEVFLGGLSSYAIVCLVVSFLQMHPRLSTSSVRQEDN


Trf5Scer   369 LGVLLIDFFELYGKNFGYDDVAISIS--DGYASYIPKSCWRTLEPS-RSKFSLAIQDPGD
Trf5Spar   369 LGVLLIDFFELYGKNFGYDDVAISIS--DGYPSYIPKSCWKTLEPS-RSKFSLAIQDPGD
Trf5Smik   369 LGVLLIDFFELYGKNFGYDDVAIGMS--DGYPSYIPKSYWKTLEPS-RSKFSLAIQDPGD
Trf5Sbay   368 LGVLLIDFFELYGKNFGYDDVAISNS--DGYPSYIPKSYWKTLEPS-RGRFSLAIQDPGD
Trf5Skud   369 LGVLLIDFFELYGKNFGYDDVAISIS--DDHPSYIPKSHWKTLELS-RSKFSLAIQDPGD
Trf5Cgla   380 LGVLLIEFFELYGKNFAYDDVAICVD--DSRATYKPKTSCRFYPPA-RTSFTLAIQDPGD
Trf5Kpol   343 LGVLLIDFFELYGKNFAYDDVAISFL--DGRTSYMPKSEFKQFQPV-RNPFSLAIQDPND
Trf5Scas   391 LGVLLIEFFELYGKNFSYDRVAISIS--NGVASYIPRKEWKDLLSN---KDRIAIQDPGD
Trf4Scer   372 LGVLLIEFFELYGKNFGYDDVALGSS--DGYPVYFPKSTWSAIQPI-KNPFSLAIQDPGD
Trf4Spar   372 LGVLLIEFFELYGKNFGYDDVALGSS--DGYPVYFPKSAWSAIQPI-KNPFSLAIQDPGD
Trf4Smik   366 LGVLLIEFFELYGKNFGYDDVALGSS--DGYPVYFPKLAWSAILPI-KNPFSLAIQDPGD
Trf4Sbay   373 LGVLLIEFFELYGKNFGYDDVALGSS--DGYPVYFPKSTWNAIQPI-KNPFTLAIQDPGD
Trf4Skud   374 LGVLLIEFFELYGKNFGYDDVALGSS--DGYPIYFPKSSWSAIQPI-KNPFSLAIQDPGD
Trf4Kpol   308 LGVLLIDFFELYGKNFGYDHVALSVM--NGKATYLPKSNWKELHPI-KSTFQLAIQDPGD
Trf4Scas   370 LGVLLIEFFELYGKNFGYDDVAISVT--DGYPSYLPKSSWKGLQQT-RGSFSLAIQDPGD
Trf4Cgla   296 LGVLLIEFFELYGKNFGYDDVAIGVQ--DGSPIYMAKRSWKSLEQN-RGSFNLAIQDPGD
TrfKlac    384 LGVLLIDFFELYGKNFGYDDVGISVS--EHEARYIPKNEHPDLSAG-RPRFSLLIQDPGD
TrfAgos    388 LGVLLIDFFELYGKNFGYDDVGIAVS--EGDASYINKKEYPELTRNLRGTFNLVIQDPGD
TrfZrou    294 LGVLLIDFFELYGKNFGYDDVAVVVA--DQRPCYMHKSDWLDLRLSGGGSFTLAIQDPGD
TrfKthe    362 LGILLIDFFELYGKNFGYDHVAISVT--DSGARYLSKELYGSLQNP-RTSFALAIQDPGD
TrfKwal    361 LGILLIDFFELYGKNFGYDHVAIAVS--DTGARYLPKQFHASMQHP-RTSFALAIQDPGD
TrfSklu    408 LGVLLIDFFELYGKNFGYDDVAISVQ--DGQVGYLPKDECPALQNP-RSTFSLAIQDPGD
TrfDhan    365 LGSLLIEFFELYGRNFSYDNLIIAIDTETDLPKYLLKRKHPVLNTS-RNPFSIIIQDPSD
TrfCgui    361 LGSLLIEFFELYGRNFSYDNLILAIDSRTDMPKYLLKRHYPELNAS-KNPFSIVIQDPSD
TrfClus    351 LGTLLIEFFELYGRNFSYDHLIVALANGED-PRYLLKSRNHVLTAG-RGQFSIVVQDPSD
TrfCtro    360 LGVLLIEFFELYGRNFSYDDLVLALDPENEEPKYVRKSKSPLQHSA-RSSFAIIIQDPAD
TrfCalb    362 LGVLLIEFFELYGRNFSYDNLILSLDRDTEEPRYEKKGNHPILNTA-RNTFSIVIQDPAD
TrfCpar    354 LGVLLIEFFELYGRNFSYDNLIISIDPETQLPRYLLKGRHPILSTA-RNTFSIVVQDPAD
TrfLelo    416 LGVLLIEFFELYGRNFSYDNLIISLDPKTEEPRYLPKGRYSVLNTA-RNTFAIVVQDPAD
TrfYlip    442 LGVLLIEFFELYGVNFNYERVALNMD---GNCGYLSKRLYPDLIGR--NPFAVAIRDPDD
TrfSoct    428 YGVLLLELLELYGKRFHYDAVGIAVH---NGGFYFSKKKQGWLRPN--QPYLLSIMDPVE
Cid14Spom  435 FGVLLLEFLELYGKQFYYDAVGIAVH---NGGFYFSKKKMGWLKPN--QPYLLSIQDPVD
TrfSjap    426 LGVLFLEFLELYGKRYNYDAVGIAVH---NGGFYFSKKKMGWVKPS--QPYLLSIQDPVD
```

```
Trf5Scer   426 PNNNISRGSFNMKDIKKAFAGAFELLVNKCWELNSATFKDRVG-----KSILGNVIKYRG
Trf5Spar   426 PNNNISRGSFNMKDIKKAFAGAFELLVNKCWELDSATFKDRVG-----KSILGNVIKYRG
Trf5Smik   426 PSNNISRGSFNMKDIKKAFAGAFELLTNKCWELDSATFKDRVG-----KSILGNVIKYSG
Trf5Sbay   425 PNNNISRGSFNMKDIKKAFAGAFELLVNKCWELDSATFKDRLG-----KSILGNVIKYRG
Trf5Skud   426 PNNNISRGSFNMKDIKKAFAGAFELLVNKCWELDSATFKDRVG-----KSILGNVIKYRG
Trf5Cgla   437 PTNNLSRGSFNVRDIKKSFSGAFDLLVNKCFELDAATFKNRVG-----KSILGNVIKYRG
Trf5Kpol   400 PYNNISRGSFNLRDIKKAFSGAFDLLTNVCFEMDFATFKDRVG-----RSILGNVIKYRG
Trf5Scas   446 PSNNIARSSKNLQGIRKAFSSAFDLLVNQCFELDTATFKDRVG-----KSILGNIIKYRG
Trf4Scer   429 ESNNISRGSFNIRDIKKAFAGAFDLLTNRCFELHSATFKDRLG-----KSILGNVIKYRG
Trf4Spar   429 ESNNISRGSFNIRDIKKAFAGAFDLLTNRCFELHSATFKDRLG-----KSILGNVIKYRG
Trf4Smik   423 ESNNISRGSFNIRDIKKAFAGAFDLLTNRCFELHSATFKDRLG-----KSILGNVIKYRG
Trf4Sbay   430 EANNISRGTFNIRDIKKAFAGAFDLLTNRCFELHSATFKDRLG-----KSILGNVIKYRG
Trf4Skud   431 ESNNISRGSFNIRDIKKAFAGAFDLLTNRCFELHSATFKDRLG-----KSILGNVIKYRG
Trf4Kpol   365 ATNNISRGSFNIRDIKKAFAGAFDLLTNRCFELHRAKLKDRQG-----CSILGNVIKYRG
Trf4Scas   427 ETNNISRGSFNLRDIKKGFAGAFDLLTNRCFELDQTSFKQRVG-----KSILGNVIKYRG
Trf4Cgla   353 VTNNISRGSFNLRDIKKAFAGAFDLLTNKCFELNSISIKERYG-----KTILGNIIKYHG
TrfKlac    441 PSNNISRGSFNLRDIKKGFAGAFDLLANRCFELDSATFKDRIG-----SSILGNVIKYKG
TrfAgos    446 PANNISRGSFNIRDIKKAFAGAFELLTNRCFELDAATFKHRVG-----KSILGNVIKYRG
TrfZrou    352 SSNNISRSSFNLRDIKKAFAGGFDLLTNRCFELDAATFKDRVG-----KSILGNVIKYRG
TrfKthe    419 KDNNISRGSFNIRDIKKAFAGAFDLLTNRCFELDAATFKDRVG-----KSILGNVIKYRG
TrfKwal    418 KENNISRGSFNIRDVKKAFAGAFDLLTNRCFELDAATFKDRVG-----KSILGNVIKYRG
TrfSklu    465 SSNNISRGSFNLRDIKKAFAGAFDLLTNKCFDLDAATFKDRLG-----KSILGNVIKYRG
TrfDhan    424 PANNITRSSYNLRDLKKAFGGAFQLLVDKCYELNGASYKSRIG-----QSILGDIIKYRG
TrfCgui    420 PSNNISRSSYNLRDVKKAFGGAYQILIDKCYELHSATYRARIG-----QSILGDIIKYKG
TrfClus    409 PANNITRSSYNLRDLKKAFGGAYQLLVEKCYELHAASYKNRIG-----QSILGDIIKYRG
TrfCtro    419 PSNNITRSSYNLRDLKKAFGGAYQLLSEKCYQLNAASYKERLG-----SSILGDIIKYTG
TrfCalb    421 TTNNITRSSYNLRDLKKAFGGAYQLLVEKCYQLNAASYKQRLG-----QSILGDIIKYNG
TrfCpar    413 SSNNITRSSYNLRDLKKAFGGAYQLLVAKCYELNAAPYRERLG-----KSILGDIIKYKG
TrfLelo    475 PNNNITRSSYNLRDLKKAFGGAYQLLVAKCYELHAASYRARLG-----QLILGDIIKYKG
TrfYlip    497 HENNISQGTFNMRNIKRAFSGAFQLLSGKCLDMNKTGYKGRQG-----KSLLTSIIKLKG
TrfSoct    483 FSNDVSKSSRGLLRVKATLGNGFDLLTNELFALASRIEREGSRRISDFPSILGSIISVDE
Cid14Spom  490 FQNDVSKSSRGLLRVKATFANGFDLLTSKLYALASRIEREGVNRVKDFPSILSTILSVDE
TrfSjap    481 YDNDISKSSRGILRVKATLGNSFDLLTSELYSLAARIEREGLHILKDEKSLLSCIIGIDD


Trf5Scer   481 QKRDFNDERDLVQNKAIIENERYHKR-RTRIVQEDLFINDTEDLPVEEIYKLDEPAKKKQ
Trf5Spar   481 QKRNFKDERDLVENKAITENERYHKR-RTRIIQEDLFINDIDELPVEEIYKLDEPLKKKR
Trf5Smik   481 QTRDFKDERGLVENKAIIENERYHKR-RTRIVHEDVFINDTEDLPAEEIYKLEEPPKKKR
Trf5Sbay   480 QQRDFKDERDLVENKAILENERYHKK-RTRIVHEDMFINDTEDLPVEDIYKLDEPLKKKR
Trf5Skud   481 QKRDFKDERDLVENKAIIENERYHKR-RTRIVHEDVFINDTEDLPAEEIYKLDEPPKKKR
Trf5Cgla   492 APRDFNDERALVENRAIIENEHYHRK-RARVVHAD--------DEDTSDEDDMF-INISA
Trf5Kpol   455 EKRDFKDERDLIENAAILTNEEYHRK-RSRIVHEDIFLDTTDSEPDLDEEQDMYHIDIPV
Trf5Scas   501 AMRNFRDERHLVVNRAIKENENFYRE-LEDKKRKEKAEKEEMFLNPSSDEAELDEALLGE
Trf4Scer   484 KARDFKDERGLVLNKAIIENENYHKK-RSRIIHDEDFAEDTVTSTATATTTDDDYEIT--
Trf4Spar   484 KARDFKDERGLVLNKAIIENENYHKK-RSRIIHDEDFTEETATSTATATTTDDDYEIT--
Trf4Smik   478 KARDFKDERGLVLNKAIIENENYHKK-RSRIIHDEEFTEDTVTSTAT----DDDYEII--
Trf4Sbay   485 KARDFKDERGLVLNKAIIENENYHKK-RSRIIHDEEFAEDIVTSTATASTTEDDYDII--
Trf4Skud   486 KARDFKDERGLVLNKAIIENENYHKK-RSRIIHDEEFTEDTATS--TATTTEDDYEII--
Trf4Kpol   420 KARDFRDERGLVENKAIIENENYHKR-RSRIIHGSDNEEEEEEE--ESDKEDDPYNIE--
Trf4Scas   482 KERDFKDERSLVVNKAIVENEKYHKK-RSRIVHKVNPDEVSQTVEVVADEDDGDDMYIVE
Trf4Cgla   408 KRRDFRDERGLVQNKAIAENESYHKK-RSIIMH----GELSAVNGKRSADDESN------
TrfKlac    496 TKRDFNDERDLVINEAIQENEKYHNK-RGRIVHDDLF----TDISDIEAEEEIYQVQEPP
TrfAgos    501 AKRDFKDERALIVNKAIQENEEFHQR-RGRIVHRDTA----EFIN--ISDDDDYELQPPP
TrfZrou    407 KLRDFNDDRDLVANSAIIENEKYHKK-RSRIVHDNDE----VFIDPSESENE--------
TrfKthe    474 KQRDFKDERNLVVNKAIAENETYHRK-RGRIVHEEVF----TDVSDGEKEDDIYTVEEP-
TrfKwal    473 KQRDFKDERDLVVNRAIAENEKYHRK-RGRIVHQEVF----TDASDGEKEDDIYTVAEP-
TrfSklu    520 KQRDFKDERSLVINKAIVENEQYHKK-RGRIVKGEIF----TDVSDNENEEDLYKVEGP-
TrfDhan    479 KERDFNDDRHKVTNDALINHENEEGN--DDDESDGANGNNKYYFSDMTVESEEDTTFA--
TrfCgui    475 KERDFNDERHMVVNQAMVTDDPTPAI--TDDESDGHNGNDKYYFSDMTVDSEDDTFTAN-
TrfClus    464 KDRDFNDDRDKVVNSALISHLDDE----DDDESDGADGNDKYYLSDMTSE-EEETYVAP-
TrfCtro    474 KERNFSDDRHKVVNHALVKHDDDDDGQEDDAESDGQNGNDKYYFSDMTDESDE-PYVPSV
TrfCalb    476 KERDFNDDRHKVVNHALISHEEEIDD-----ESDGLDGNDKYYFSDMTVESNEDEYVP--
TrfCpar    468 KERNFVDERHLVRNDALIHHEEHPNT-EQDLESDGLEGNDKYYFSDMTVESEE-EYTPLP
TrfLelo    530 KERHFRDERHMVVNEALISHEEETTS--LDGKKIVTKSNDEFYFSDMTDSDETEEYKPQL
TrfYlip    552 PVRDQTPDAITEPLKYTGGGDEFKIP--QDAVYNSVLMNDEDEDEKAKQAEDADKTKRRA
TrfSoct    543 GVRKHRVHMLNCFNEHPIALEPMVDVEQLSSIDVDRLPTENVDLQFVEDESDSDEVEQET
Cid14Spom  550 GVRQHREHMLKCYKNNPVPLEPLVEVDALASIDVDKLPLQDVGLQYVEDESDSDETDAAK
TrfSjap    541 NITKHREHIQQCYACDPVPLEPLTSIQNLTSIDVDAVQLPAMELEFVEGESESDDVAEVA
```

```
Trf5Scer   540 KAKKDKREGE-------------------IKKSAIPSPPPDFGVSRSKLKRKVKKTDQGS
Trf5Spar   540 KVTKDKRKGR-------------------IKKSAIPSPPPDIAVSKRKIQGKVKEVDHGS
Trf5Smik   540 KATKHKREGK-------------------TKKSAIPSPPPELAVNKNKRQLEKKDTGPGS
Trf5Sbay   539 KGKKDKKERK-------------------TKERTVPSPASEAAVKKSKRQHQKKAADLSP
Trf5Skud   540 KAKKDKKERS-------------------TKESAIPSPPPELTVTKTKRKHKKKTVTAGS
Trf5Cgla   542 NEE-ETEEMY---------------------KVEKVEKVVKKR-KIAKADTNTKTKTKTK
Trf5Kpol   514 PKKRKVQKER---------------------TINPAILLAQKRSKLKSSKSDSKSEKPNK
Trf5Scas   560 RAEDIYKLDR--------------------PLKRTKNDKHKHKHKHKHTNTDKNTKKEKV
Trf4Scer   541 -------------------------------------------NPPAKKAKIEEKPE---
Trf4Spar   541 -------------------------------------------NPPTKKAKIEAEPK---
Trf4Smik   531 -------------------------------------------RPPAKKAKMEVLEPGFI
Trf4Sbay   542 -------------------------------------------EPPTKKAKTKFSEPEAK
Trf4Skud   541 -------------------------------------------EPPTKRARTETVKPQPK
Trf4Kpol   475 -------------------------------------------EPPMKKLKRSSSIK---
Trf4Scas   541 -------------------------------------------EPPSKKRKKSSNKA---
Trf4Cgla   457 -----------------------------------------------EHHDDTQNKK---
TrfKlac    551 TKKTKKDKSKEKKERKEKRREKRKKQQLKENTERVQEVEEDNYDPTEAIIVNDEPSEQED
TrfAgos    554 -----------KRAR----------------------VPAPAPAPADNVVVLDDP---AD
TrfZrou    454 -----------------------------------------ALKEEQDSYLIQEPEQPPK
TrfKthe    528 --------------------------------------------AKKKPKKAKKAKKAKK
TrfKwal    527 --------------------------------------------AKKKSKKAKKAKKAKK
TrfSklu    574 --------------------------------------------ARKKKK--KKTQKVKK
TrfDhan    535 --------------------------------------------------------QEIK
TrfCgui    532 -------------------------------------------------------KKEVS
TrfClus    518 -------------------------------------------------------EHKQP
TrfCtro    533 --------------------------------------------AKPTP------TPLTK
TrfCalb    529 --------------------------------------------EKKRT------TPVTN
TrfCpar    526 --------------------------------------------TKPTLVSKQ--TPITN
TrfLelo    588 --------------------------------------------LKVKSHKTISPTPVTN
TrfYlip    610 AQREEQKKKK--------------------ELTTASDDKIPKGPAADRNQPKADQRKMSK
TrfSoct    603 DDLIKK-------------------------------------KGLNDLQNTNHN-EGLS
Cid14Spom  610 DDLFKVNESI-------------------------------ETNGHENFQKQALTSTGEQ
TrfSjap    601 APSKIAN-------------------------------------------RMIHSIENEK


Trf5Scer   581 LLHQNNLSIDDLMGLSENDQESDQDQKGRD--------------------TPSGQDEKSP
Trf5Spar   581 PPHQKNLSIDDLMGLSENDQESDQDQKEKD--------------------TPPGQDEEPP
Trf5Smik   581 LPHHNNLRIDDLMGLSENEEESSHDQKESD--------------------ISFGQDGKPP
Trf5Sbay   580 IPHHDNPSIDALMGLSDNDEGSDQDRRGSG--------------------IQPGQDGKQP
Trf5Skud   581 FTQRGNLSIDALMGLSDNDEGSGQDQRGSE--------------------VSLGQDEKQP
Trf5Cgla   579 AKARNHTSIDQLMGIPEDEEPPVIQERE--------------------------------
Trf5Kpol   553 KKQLPDKFIDSLMGIVSDDEQEEGEEGKNSIKQKISGHSRSKDNKKYETTTDYNSDEEKI
Trf5Scas   600 EKVKTNLKVAKSQSIITSKNRTIDILKGLAKG-----------TNTKISGDEMIIDESTA
Trf4Scer   555 SEPAKRNSGETYITVSSE-DD---------------------------------------
Trf4Spar   555 IEPAKEESGETHIIISSE-DDD--------------------------------------
Trf4Smik   548 SEPSKEKSGETHITISSENDDD--------------------------------------
Trf4Sbay   559 KEPLKEKSEETYITISSGDDDC--------------------------------------
Trf4Skud   558 SEPSEKKSEESHITISSDDDDDDDE-----------------------------------
Trf4Kpol   489 EDDGNAKKKPRKERNRRQKEDQD-------------------------------------
Trf4Scas   555 EKIKKKKTVDSYMGLPSE------------------------------------------
Trf4Cgla   467 QKVN-----------DYD------------------------------------------
TrfKlac    611 DYDPSKPSVDDYMGLPEENNDENDKDTKEEARTPV------INLIEDENGASDDSNSDPK
TrfAgos    578 DYVPAQP-VDKLMGLDDT---------------------------DDYLPAPASPTDTPK
TrfZrou    473 KRRQTKS-VDELMGVPNEDEE------------------------DDYNPLNRETDESIT
TrfKthe    544 ETKASSSSVDTLMGLDTQDDADEDGYDPSN---------------EAPSTLALEDARSDI
TrfKwal    543 DPKPSTKTMESLMGLE---ESDDDGYDPSN---------------EAPATLVSEDSKSDI
TrfSklu    588 D---NAKVVNQLMGIN----DEEDDYNPVQ---------------TNMNEEEGDDYNPEE
TrfDhan    539 PEDPNKKKVADFMSINVPSED-----EEDDPKE---------------KKEEDTETQDSD
TrfCgui    537 AKD-NEEKISGLLGIKNENE-----------------------------ADEVSETNEDD
TrfClus    523 QKNENKASVESFMGIQ-------------------------------------SESEEEE
TrfCtro    543 KPKDTKKLIESMLSLNNSSD------EDKGSKE---------------ENEEYVPTVGTK
TrfCalb    539 KAKDTKKLVESFLSLEDSSDVEELSAKDVAGKF---------------EDEPYIPKL--K
TrfCpar    540 KAKDTKKLVESYLSLESSPEAQEKTRDDDAAAAPA-------------TTAATEPKKPKL
TrfLelo    604 KAKDTKKLVESFLGLEKSED------ENNVNND---------------DLEYDPEKLLPP
TrfYlip    650 DRAAARAAAREVMEISSDDDDGSDSKNGGAKSN----------------PIDLSSDEEKG
TrfSoct    625 NVSDARMNAKDLFNIDEESSEEY-----------------------------EKNDNDFK
Cid14Spom  639 SSSNSRANPSKLFNISSDDSE---------------------------------DEVPII
TrfSjap    618 N--ESAYSSQEFFSMNEASFDDG-----------------------------NSPENAVL
```

```
Trf5Scer   621 LETKTVDAQTRRDYWLSKGQAL-------------
Trf5Spar   621 LETKTVDAQTRRDYWLSKGQAL-------------
Trf5Smik   621 LETQTVDAQTRRDYWLSKGRAL-------------
Trf5Sbay   620 LKTQTVDAQTRRDYWLSKGQAL-------------
Trf5Skud   621 LELQTVDAQTRRDYWLSKGQAL-------------
Trf5Cgla   607 --KKSLSMQDKRDYWLSKGQAL-------------
Trf5Kpol   613 ELKKKVDAQTRRDYWLSKGQSMSTIATT-------
Trf5Scas   649 LQNTKVDAQTRRDYWLSKGHVLSGTGGLGPEKTTV
Trf4Scer   576 ------DEDGYNPYTL-------------------
Trf4Spar   577 ------DEDGYNPYTL-------------------
Trf4Smik   571 ------DEDGYNPYTL-------------------
Trf4Sbay   581 ------DEDGYNPYSL-------------------
Trf4Skud   583 ------DEDGYNPYSL-------------------
Trf4Kpol   512 ------DEEEEDDYNPLKR----------------
Trf4Scas   577 ------EKEDEDDYNPTLLR---------------
Trf4Cgla   478 ------DAEEEDDYDPLA-----------------
TrfKlac    665 MQ--LVDKQTKRDYWLSKGQAM-------------
TrfAgos    610 L-----DKAAKRNYWLSKGQTM-------------
TrfZrou    508 LKNFTVDAQTKRDYWLNKGNSMSN-----------
TrfKthe    589 RKK-SVDAQTKRDYWLSKGQAL-------------
TrfKwal    585 RKK-SVDAQTRRDYWLSKGQAL-------------
TrfSklu    626 DKKNYVDKRTKRDYWLSKGQNL-------------
TrfDhan    579 KQKNNLDKDIKRDYWRQKGLGL-------------
TrfCgui    567 KQKSNLDKNIKRDYWRQKGLEL-------------
TrfClus    546 KVRTNLDKDVKREYWRSKGLEI-------------
TrfCtro    582 KPKSNLDKDVRRDYWRQKGLDL-------------
TrfCalb    582 KSKSNLDKDIKRDYWRQKGLDL-------------
TrfCpar    587 PRKPVVDKDFKRDYWRSKGFDM-------------
TrfLelo    643 KPKSNLDKDVKRDYWRQKGLEL-------------
TrfYlip    694 RSDIKKDPSAKRRFWQQKGGLDY------------
TrfSoct    656 RDIQRSDEEESRATKVRKKTR--------------
Cid14Spom  666 EDTTASDEE-SRAKKIRKRF---------------
TrfSjap    647 VDDE--EEEESRASSLGKRRRTD------------
```

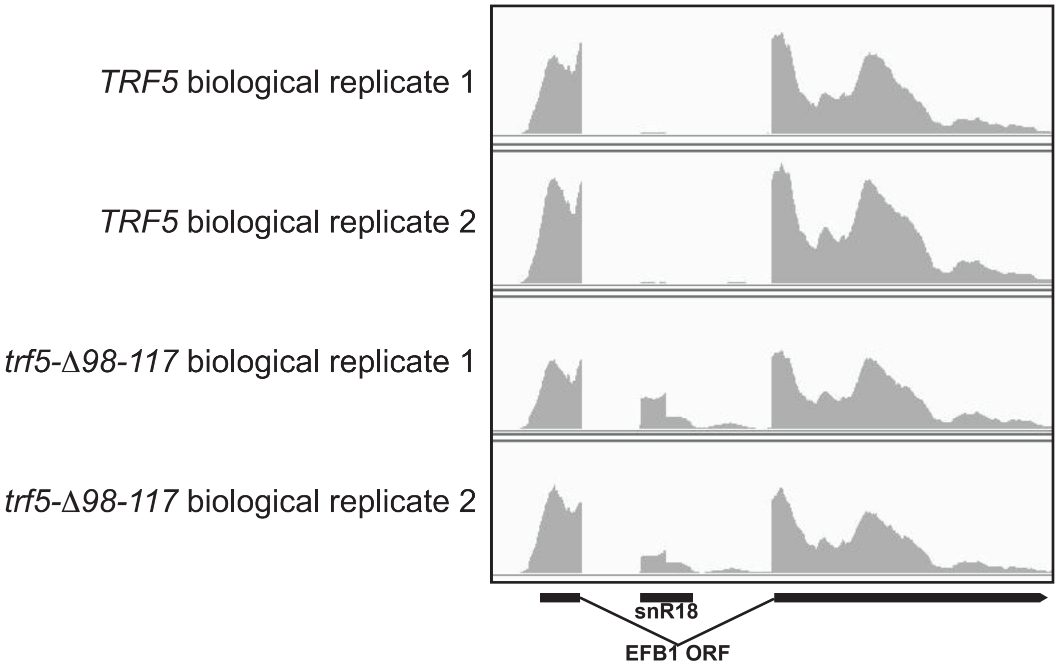

Figure S2: Transcriptome sequencing from duplicate cultures of TRF5 and trf5-Δ98-117 strains is highly reproducible. Shown is the region that includes snR18 snoRNA and EFB1 mRNA as an example.

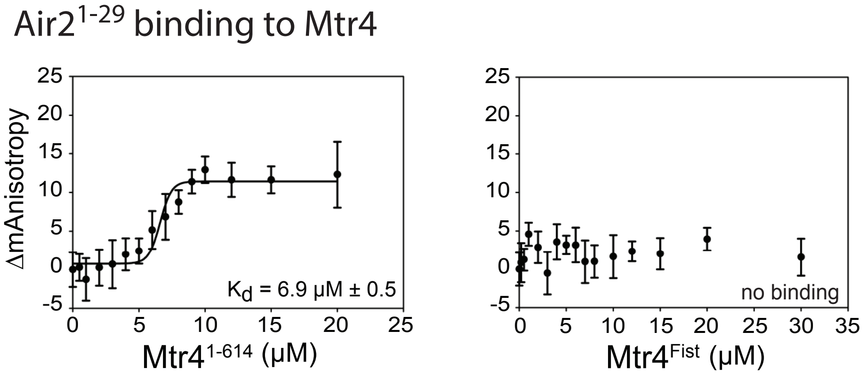

Figure S3: The Air2 N-terminal peptide binds to a truncated recombinant Mtr4 that contains only the two RecA domains, but not to the isolated fist domain. Fluorescent anisotropy measurements were performed as in figure 2.
